# Theoretical and Numerical Study of the Buckling of Axially Compressed Bimodular Thin Cylindrical Shells

**DOI:** 10.3390/ma19142964

**Published:** 2026-07-09

**Authors:** Jun-Song Ran, Xiao-Ting He, Jun-Yi Sun

**Affiliations:** 1 School of Civil Engineering, Chongqing University, Chongqing 400045, China; 202316131371@stu.cqu.edu.cn (J.-S.R.); sunjunyi@cqu.edu.cn (J.-Y.S.); 2 State Key Laboratory of Safety and Resilience of Civil Engineering in Mountain Area, Chongqing 400045, China

**Keywords:** buckling, bimodular effect, cylindrical shells, axial compression, critical load

## Abstract

Bimodular materials refer to materials that exhibit distinct elastic properties under tensile and compressive loading conditions. This study aims to analyze the buckling problem of bimodular thin cylindrical shells under axial compressive loading. In existing studies on the buckling behavior of thin shells, the bimodular effect of materials is rarely considered due to the complexity of the analysis. Therefore, based on the elasticity theory of different moduli, this paper presents a theoretical and numerical study on the buckling problem of thin cylindrical shells with bimodular effect under axial compression. Based on the deformation characteristics of thin cylindrical shells under axial compression before and after buckling, a simplified mechanical model suitable for the buckling analysis of bimodular axially compressed thin cylindrical shells is established. The simplified mechanical model proposed in this paper decouples the membrane effect and bending effect during shell buckling by introducing the concept of the physical neutral layer. It divides the shell cross-section into tensile and compressive regions according to the bending effect component, thereby fully accounting for the influence of the bimodular effect of materials on the lateral bending stiffness of the shell. Subsequently, through this simplified mechanical model, the analytical expression for the linear critical load of bimodular thin cylindrical shells under axial compression is obtained. Eigen Value buckling analysis of bimodular axially compressed thin cylindrical shells based on ABAQUS is carried out to validate the correctness of the analytical solution. The results indicate that the shell height *L*, the radius-to-thickness ratio *R*/*t*, and the tension-to-compression modulus ratio *E*^+^/*E*^−^ have significant influences on their linear critical load. Moreover, the larger the tensile to compressive modulus ratio, the more sensitive the linear critical load of the shell is to the bimodular effect of materials. Specifically, when the *E*^+^/*E*^−^ ratio of the shell equals 1/2, the corresponding linear critical load is approximately 10% higher than that predicted by the classical solution. Meanwhile, when the *E*^+^/*E*^−^ ratio equals 2, the linear critical load of the shell is approximately 21% lower than that predicted by the classical solution. The novelty of this study lies in that, for the first time, based on the simplified mechanical model of tension-compression subarea, the influence of bimodular effect of materials is considered in the buckling analysis of thin cylindrical shells under axial compression, which provides a new analytical idea for the refined analysis and optimal design on it.

## 1. Introduction

Outstanding mechanical properties under compression, bending, and torsion are conferred by the closed circumferential geometry of cylindrical shells; this geometry also permits highly efficient material usage. Consequently, such shells are widely employed in the aerospace, marine, and civil engineering sectors [1,2,3,4,5,6]. In engineering applications, these shells often serve as pressure-bearing components and are predominantly subjected to axial or radial compressive loads; as a result, the internal forces are dominated by membrane forces and bending moments. In particular, thin-walled cylindrical shells are highly susceptible to buckling failure when subjected to axially compressive loading [7].

For cylindrical shell structures, the linear critical load derived from small deflection buckling theory has long been the core index for the stability design in practical engineering, as it provides a reliable and conservative prediction of the buckling load for perfect shells. The linear buckling of thin cylindrical shells under axial compression has thus long stood as a central issue in solid mechanics research. In structural stability analysis, buckling evaluation mainly includes eigenvalue buckling and nonlinear buckling analysis. Eigenvalue buckling solves the generalized eigenvalue problem of elastic and geometric stiffness matrices, providing a conservative upper bound for perfect structures with high efficiency [8]. Nonlinear buckling traces the full equilibrium path via incremental loading, capturing nonlinear effects and postbuckling behavior but requiring more computational resources [9]. Notably, eigenvalue buckling modes are widely used as initial imperfections in nonlinear simulations [9]. This study adopts eigenvalue buckling analysis to derive the closed-form critical load solution for bimodular shells, laying a foundation for subsequent imperfection-involved postbuckling research. At the same time, progress in materials science and advances in shell fabrication methods have allowed engineers to embed high-performance composites into such shells with increasing frequency. Their efficient employment in various complex and extreme conditions is consequently made possible. However, the intrinsic anisotropy or nonlinear constitutive behavior of composites (e.g., the bimodular effect) poses substantial obstacles to theoretical predictions of buckling and failure. Hence, linear buckling studies of composite thin cylindrical shells have rapidly emerged as a primary focus within shell structure research in recent years [10,11,12,13,14,15,16].

By incorporating the bimodular effect of materials, this work offers a new extension to the buckling problem of axially compressed thin cylindrical shells. Notably, the bimodular effect exists in nearly all engineering materials, whether monolithic or composite, with only the degree of its prominence varying [17,18,19]. Systematic uniaxial tensile and compressive tests have confirmed the presence of pronounced tension-compression modulus asymmetry in typical cast metal alloys, such as zinc-aluminum (ZA) alloys and tin bronze matrices. For the widely used ZA27 cast zinc-aluminum alloy, the measured tensile elastic modulus is approximately 77.9 GPa, while the compressive elastic modulus reaches about 89 GPa, corresponding to a relative difference of 14.2%. For ZCuSn10P1 tin bronze, a representative bearing bronze matrix, the tensile elastic modulus is around 100 GPa and the compressive elastic modulus is approximately 109 GPa, with a relative difference of 9.0% [20]. This asymmetry originates from the anisotropy of grain orientations, multiphase microstructures, and the asymmetric dislocation motion under tensile and compressive loading conditions [21]. Furthermore, the bimodular effect is not exclusive to fiber-reinforced composites. It spans multiple length scales, and a large body of experimental evidence has been accumulated over recent decades. For advanced nanocomposites reinforced with carbon nanotube or graphene nanofillers, the mismatch in interfacial bonding strength and the preferential orientation distribution of nanofillers further intensify the tension-compression modulus asymmetry. With optimized filler content and dispersion state, the ratio of tensile modulus to compressive modulus can exceed 1.5, which has been validated by molecular dynamics simulations and quasi-static mechanical tests [22,23]. Meanwhile, from the perspective of tribology and surface engineering, the formation of tribologically induced layers (tribolayers), work-hardened subsurface zones and microcrack arrays during wear processes causes pronounced discrepancies in elastic properties between the modified surface layer and the bulk substrate. In advanced lubricated mechanical systems employing heterogeneous coating structures—such as diamond-like carbon films and ceramic-reinforced composite coatings—this surface-substrate modulus mismatch becomes more prominent, leading to apparent bimodular characteristics in macroscopic contact stiffness and load-bearing response [24,25]. In the field of fracture and damage mechanics, extensive studies have confirmed that internal microcracks and microvoids exhibit fundamentally different mechanical behaviors under tension and compression: cracks open under tensile stress and degrade the overall stiffness, whereas they close under compressive stress and partially restore structural stiffness. Combined with the frictional sliding effect on crack surfaces, this unilateral contact mechanism ultimately gives rise to macroscopic tension-compression modulus asymmetry. This phenomenon has been widely observed in rock, concrete and fatigue-damaged metallic materials, and has become a core basis for developing unilateral damage constitutive models [26,27].

Practical engineering has seen widespread adoption of composite cylindrical shell structures; meanwhile, for composite materials in particular, the combination of distinct material constituents typically results in a significant bimodular effect. In marine engineering, for instance, composite cylindrical shells are routinely utilized as principal load-bearing elements for the structural design of submarines and underwater vehicles; this practice stems from their superior mechanical performance and outstanding corrosion resistance [28]. Similarly, within the aerospace sector, these composite shells are widely utilized as critical load-bearing members in solid rocket motors; during launch, they primarily bear axial compressive forces and transmit thrust between rocket stages [29]. These components are required to deliver stable and reliable performance under severe and harsh operating conditions; thus, their buckling load-carrying capacity is typically subject to stringent control. In this context, linear buckling analysis incorporating the bimodular effect for composite cylindrical shells is therefore of substantial research value and engineering significance. Explicitly accounting for the bimodular effect in the analysis enables the full mechanical potential of composite cylindrical shells to be exploited, offering a theoretical reference for refined performance assessment and optimal structural design of such components. Unfortunately, the vast majority of existing investigations on shell stability assume that materials possess identical tensile and compressive moduli, a simplification that essentially neglects the inherent bimodular effect. More recently, He et al. [30] conducted a series of theoretical studies on the postbuckling behavior of bimodular axially compressed thin cylindrical shells, their research results are only applicable to the analysis of perfect shells, and did not take into account the influence of shell imperfections. To extend the research on the stability behavior of bimodular axially compressed thin cylindrical shells to a more general case, it is necessary to introduce the corresponding linear buckling analysis results as the shell imperfection, so as to fully account for the influence of the coupling effect between the material bimodular effect and different imperfection forms on the postbuckling behavior of axially compressed thin cylindrical shells. Therefore, it is necessary to conduct the corresponding analysis of the linear buckling behavior of bimodular axially compressed thin cylindrical shells to serve as an important supplement. However, in the existing research, studies on this aspect are quite scarce. To address this research gap, the present study aims to conduct a systematic theoretical and numerical study of the linear buckling behavior of bimodular thin cylindrical shells under axial compression. The remainder of this introduction is organized as follows. First, we will introduce the bimodular material model and its application in structural analysis. Next, we will present a brief historical review of the fundamental theories related to the linear stability of cylindrical shells under axial compression. Finally, we will discuss the key challenges encountered in this research, as well as the main contributions of the present study.

**Figure 1:**
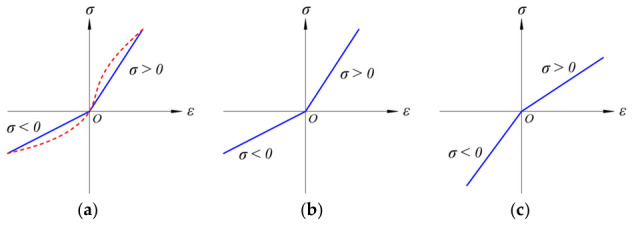
Bi-linear constitutive model for materials with bimodular effect: (**a**) Realistic behavior of materials (the red dashed curve); (**b**) $E^{+}>E^{-}$; (**c**) $E^{+}<E^{-}$ [30].

In the framework of classical elasticity theory, it is conventionally assumed that materials exhibit identical mechanical properties under tensile and compressive loading conditions. Nevertheless, this assumption is merely an idealized approximation. As discussed earlier, a vast array of engineering materials exhibit, to varying degrees, different elastic moduli in tension and compression—a behavior known as the bimodular effect. The formal concept of bimodular materials was first explicitly introduced by Jones [31] in 1977. For mechanical analysis of structures or components fabricated from such materials, the principal task lies in establishing and selecting suitable constitutive models that accurately capture the material properties. In the existing literature, two main categories of constitutive models are widely employed, and the essential difference between them resides in the criterion adopted to distinguish between the tensile and compressive moduli. One of them is Bert’s model [32], which was originally developed for anisotropic materials. This model takes the sign of the longitudinal strain of fibers as the judgment criterion, and it is commonly applied in the analysis of composite laminate structures [33,34,35,36]. The other one is Ambartsumyan’s model [20], which was proposed for isotropic materials. In this model, the sign of the principal stress at a specific material point is used as the judgment criterion. This model describes the stress–strain relationship of the material as a piecewise function at the origin, as illustrated in Figure 1. Specifically, to differentiate between the tensile modulus and the compressive modulus, the tensile modulus *E*^+^ is adopted when the stress *σ* is positive (i.e., the material is in a tensile state), while the compressive modulus *E*^−^ is used when the stress *σ* is negative (i.e., the material is in a compressive state). For conventional materials where the bimodular effect is not considered, the two moduli satisfy the relationship *E*^+^ = *E*^−^, and thus the bi-linear model shown in Figure 1 can be simplified into a conventional straight-line stress–strain relationship. Figure 1a presents a dashed curve that represents the true stress–strain relationship of bimodular materials, suggesting that the actual curve remains smooth and continuous at the origin. Wolf’s experimental findings [37] further confirm this observation. However, incorporating the smoothness of the stress–strain curve at the origin renders the entire analytical treatment exceedingly complicated, while offering negligible practical value for engineering applications. For this reason, in practical engineering analysis, the bi-linear constitutive model is the most commonly used approach to describe the bimodular properties of materials.

During the past two to three decades, the growing attention paid to the mechanical properties of materials, together with the continuous progress in mathematical solution techniques, has promoted a relatively active research period for the application of bimodular material models in structural analysis. During this period, a large number of analytical and numerical research works have emerged, most of which are based on the Ambartsumyan model. One core requirement of this model is that the stress state of each material point needs to be determined in advance before the analysis. Such a preliminary evaluation is feasible only for certain structural elements under simple loading conditions—for instance, beams or plates in pure bending—where the stress states at material points can be reasonably estimated before analysis [38,39,40]. In more complex practical scenarios, structures typically experience complicated stress states, rendering direct application of the Ambartsumyan model to obtain analytical solutions extremely difficult. To address this limitation, the finite element method (FEM) combined with iterative strategies has been widely employed in the relevant literature. In the widely adopted direct iterative variable-stiffness method, the principal stress state of each element is reassessed at every iteration, so that the stiffness matrix can be updated for the subsequent iteration [41,42,43]. Driven by the convergence difficulties inherent in these iterative methods, Du et al. [44] proposed a new computational framework capable of achieving asymptotically quadratic convergence for both small- and finite-deformation problems. Studies on the effects of bimodular materials have progressively moved beyond conventional structural elements such as beams and plates [38,39,40], and now extend to more intricate structures including arches and shells [45,46]. In addition, this research has also been extended to the field of road engineering materials, including cement-stabilized base layers [47] and asphalt pavements [48]. Due to these developments, the research on the bimodular effect has been attracting increasing attention from the international academic community.

Within the domain of solid mechanics, the linear buckling of axially compressed thin cylindrical shells has historically stood as a classic problem. The earliest research work in this field can be traced back to the pioneering studies by Lorenz [49] and Timoshenko [50]. Back in the early 20th century, these two researchers developed the classical linear small deflection buckling theory, which provided the complete theoretical formulations for the critical buckling load of thin cylindrical shells under uniform axial compression. In this theoretical framework, the geometric nonlinearity caused by large deformation was not taken into account. In addition, the geometric relationship of shell deformation was simplified by neglecting the nonlinear terms induced by the rotation of the shell mid-surface. Based on these simplifications, they successfully derived a closed-form analytical solution for the critical buckling load of axially compressed cylindrical shells. By this stage, the core framework of shell stability theory had largely been established. Later experiments by Robertson [51], however, revealed that the actual critical load of axially compressed thin cylindrical shells was far lower than the predictions of Lorenz and Timoshenko—in some instances, only one quarter of the theoretical value—and the measured data showed substantial scatter. This pronounced discrepancy between theory and experiment drew extensive research interest, and a surge of investigations into the linear stability of such shells consequently emerged during this period, all aimed at refining the small-deflection buckling theory.

In recent years, researchers have continuously refined the small deflection linear buckling theory of cylindrical shells. For instance, Ji [52] proposed a refined linear-elastic solution for the elastic critical buckling of axially compressed thin-walled cylindrical shells, which re-examined the circumferential closed-loop eigenmode solution of non-axisymmetric periodic buckling, providing a new perspective to understand the discrepancy between classical theoretical predictions and experimental results. Meanwhile, Musa et al. [53] derived a closed-form formula for the non-axisymmetric buckling stress of axially compressed circular cylindrical shells, which fully considered the influence of geometric and material parameters, and provided a convenient design tool for engineering practice. In addition, Tănase et al. [16] conducted an analytical and numerical study on the linear buckling behavior of FRP-reinforced steel cylindrical shells under axial compression, based on the Donnell small deflection shell theory, which verified the effectiveness of the small deflection theory in the stability analysis of composite reinforced shells. In addition, Civalek [54] applied the discrete singular convolution method to the linear buckling analysis of composite cylindrical shells, which provided an efficient numerical solution for the small deflection buckling problem of composite shells. Panek et al. [55] conducted experimental tests on the buckling of composite cylindrical shells under multiaxial loading, which provided experimental verification for the small deflection buckling theory. Shen et al. [56] investigated the strain response and buckling behavior of composite cylindrical shells under external pressure, which verified the effectiveness of the small deflection theory for different boundary conditions.

**Figure 2:**
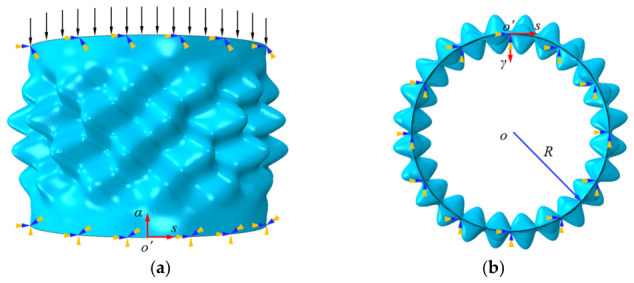
Buckling failure model of a thin cylindrical shell under uniformed axial compression: (**a**) Front view of the shell model; (**b**) Top view of the shell model.

Prior to commencing the formal research work, the primary and foremost task for us is to establish a corresponding mechanical model that can provide solid support for the subsequent theoretical analysis. Specifically, within the framework that combines classical bending theory and membrane theory, a criterion is required to establish whether the stress at a given material point along the three mutually perpendicular directions (axial, circumferential, and radial) is tensile or compressive. For illustration, the linear buckling mode of an axially compressed thin cylindrical shell—which generally assumes a periodic, small-amplitude diamond deformation pattern—is presented in Figure 2. Obviously, if we only take the membrane effect into account, the axial stress can be directly determined as compressive. However, ascertaining whether the circumferential stress (along the *s*-axis in Figure 2) and the radial stress (along the *γ*-axis) are tensile or compressive poses considerable difficulty. This is because the deformation of the shell is quite complicated, which involves both outward bulging and inward concave deformation along the radial direction at the same time. What is more, in this case, we also need to take the bending effect into full consideration. Hence, ascertaining the stress state and reducing the mechanical model emerge as a problem of considerable difficulty, and this represents the critical issue that must be tackled first in this investigation.

The buckling behavior of axially compressed thin cylindrical shells exhibiting the bimodular effect is investigated theoretically and numerically in this study. From the potential bimodular properties of advanced materials, this study focuses on their effects on the mechanical behavior of engineering structures. Compared with classical investigations into bimodular shell buckling, the proposed tension-compression partitioned model delivers a closed-form critical load solution with improved engineering practicability. A dedicated UMAT subroutine for eigenvalue buckling analysis of bimodular materials is developed and systematically validated through parametric studies, filling the research gap in the linear buckling analysis of bimodular axially compressed cylindrical shells. The findings of this research can provide theoretical reference and support for the optimal design of composite pressure-bearing shells in aerospace and marine engineering. The structure of the paper is as follows. Section 2 proposes a simplified mechanical model with tension-compression subarea, and based on the deformation characteristics of the shell before and after linear buckling, we determined the tension-compression state of its circumferential membrane stress. Section 3, based on the simplified mechanical model proposed in this paper, presents the basic equations of bimodular thin cylindrical shells under axial compression, including the geometric equations and physical equations, and determines the calculation formula of the neutral layer during shell buckling. Section 4, through the linear buckling governing equations of the shell, obtains the expression of the linear critical load of bimodular axially compressed thin cylindrical shells. Section 5 presents specific numerical examples, and conducts the corresponding numerical simulation based on ABAQUS. In Section 6, the influence of factors such as material properties and geometrical dimensions on the buckling behavior of the shell is discussed in detail. Finally, Section 7 presents the concluding remarks.

## 2. Theoretical Analysis Model of Bimodular Thin Cylindrical Shells

**Figure 3:**
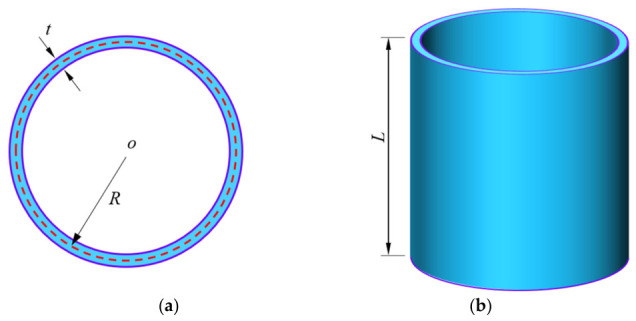
Solid model of bimodular thin cylindrical shells: (**a**) Top view of the shell; (**b**) Front view of the shell [30].

This section first presents the geometric dimensions of the solid model of thin cylindrical shells fabricated from bimodular materials, as depicted in Figure 3. For this solid model, its axial length is denoted by *L*, the radius *R*, and the wall thickness by *t*. Since the research object of this paper is thin shells, the ratio of wall thickness to radius must strictly satisfy *t*/*R* ≤ 0.05. Following Donnell’s thin shell theory [57], the normal stresses and strains through the thickness are omitted when the geometrical and physical equations for the shell are derived. The geometric mid-surface of the shell wall is indicated by a red dashed line in Figure 3, that is, the *R* adopted in this paper represents the mean radius of the shell. It is worth noting that when the bimodular effect of the material is taken into account, the position of the physical neutral layer does not coincide with this geometric mid-surface.

### 2.1. Establishment of Simplified Mechanical Models

**Figure 4:**
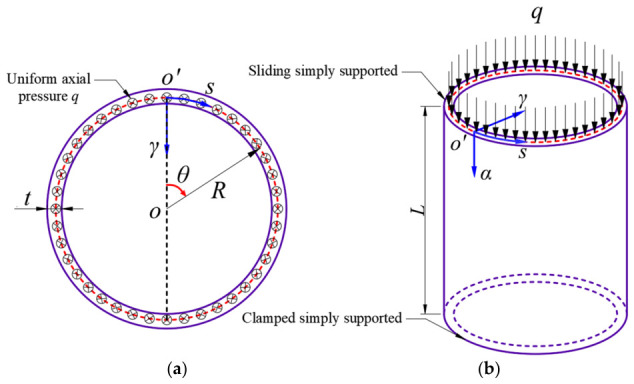
Mechanical analysis sketch of the axially compressed thin cylindrical shell under simply supported boundary condition: (**a**) Top view of the shell; (**b**) Front view of the shell [30].

Figure 4 presents the mechanical analysis sketch of a thin cylindrical shell subjected to uniform axial compression *q* (denoted by ⊗ in the top view), where the definitions of *L*, *R*, and *t* remain consistent with those in Figure 3. An orthogonal curvilinear coordinate system (o-*αsγ*) is set at the top of the shell, with the *α*-, *s*-, and *γ*-axes corresponding to the axial, circumferential, and radial directions. The local coordinate origin is *o’*, *θ* is the angle measured along the *s*-axis from *o’* to any point on the circumference, satisfying *ds* = *Rdθ*. Meanwhile, a sliding simply supported boundary condition is applied to the top of the shell, which restricts all displacements and rotations of the shell except for the radial rotation and the translational motion along the *α*-axis. A clamped simply supported boundary condition is imposed on the bottom of the shell, which restricts all displacements and rotations of the shell except for the radial rotation.

Experimental observations confirm that, for isotropic simply supported thin cylindrical shells under axial compression, the linear buckling mode consistently adopts a periodic small-amplitude wrinkling pattern, irrespective of whether the material is monolithic or composite [58,59,60], as depicted in Figure 2. Therefore, it can be inferred that when we take the bimodular effect of the material into account, the main buckling deformation mode of a thin cylindrical shell under uniform axial compression should also present a series of small alternating internal and external deflections along the *γ*-axis. In the classical stability theory of shells [49], the deflection function *w*(*α*, *s*) describing this deformation mode is set as

(1) $$w(\alpha ,s)=f\sin\frac{m\pi \alpha }{L}\sin\frac{ns}{R},$$

in which, *f* is the amplitude of the deflection function, *α* and *s* are the axial and circumferential coordinates of a point on the shell, respectively, and *m* and *n* denote the buckling half-wave numbers along the axial (*α* direction) and circumferential (*s* direction) directions of the shell during buckling, respectively. For example, *m* and *n* in Figure 2 are 4 and 24, respectively.

Additionally, for the small deflection theory of thin plates, it is generally unnecessary to consider the mid-surface deformation of the plate caused by bending and the corresponding membrane stress. However, for thin shell structures, according to Donnell’s shell theory [57], the mid-surface deformation of the shell must be considered in both the small deflection theory and the large deflection theory of shells. That is, once buckling occurs, the total stress on the cross-section can be decomposed into the bending stress induced purely by flexural deformation and the membrane stress induced purely by neutral-surface deformation. If only the bending deformation is considered, the tensile zone and compressive zone in the simplified mechanical model can be determined according to the direction of the bending moment applied on the cross-section.

**Figure 5:**
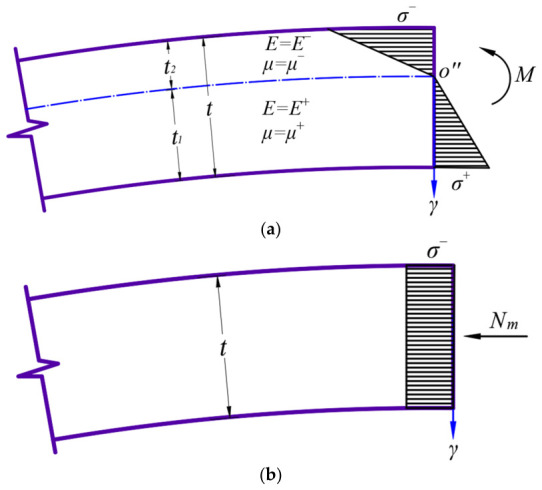
Tension-compression subarea simplified mechanical model of the shell: (**a**) Under bending moments; (**b**) Under membrane force in pure compressive zone [30].

Accordingly, a simplified mechanical model for the thin cylindrical shell exhibiting bimodular effects is constructed based on this deformation theory, as shown in Figure 5. Without loss of generality, Figure 5a displays a segment with initial curvature, taken from the shell along the circumferential direction. In this segment, the origin *o*″ of the local coordinate system lies on the neutral axis, whose location remains to be determined. Here, *t*_1_ and *t*_2_ denote the thicknesses of the tensile and compressive zones, respectively, obeying *t*_1_ + *t*_2_ = *t*, where *t* is the total cross-sectional thickness, namely the shell wall thickness. Moreover, in Figure 5, the physical quantities in the tensile zone are identified by the superscript “+”, e.g., the elastic modulus *E*^+^ and bending stress *σ*^+^, while those in the compressive zone are identified by “−”, e.g., *E*^−^ and *σ*^−^. All subsequent analyses in this paper follow this notational convention.

Clearly, in the tension–compression subarea model shown in Figure 5a, the local coordinate origin *o*″ is placed on the physical neutral surface, rather than on the conventional geometric midsurface. This coordinate choice effectively eliminates the coupling stiffness that would otherwise arise from the combined action of bending moment and membrane force on the cross-section. Consequently, bending deformation and mid-surface deformation can be handled as two independent processes, substantially simplifying the subsequent derivation of the governing equations. It should be emphasized that this is merely a change of reference frame, and thus it does not fundamentally alter the intrinsic mechanical behavior of the physical system. Strictly speaking, the tensile or compressive modulus at any point within the shell should be determined by the actual total stress state at that point. For buckling of axially compressed thin cylindrical shells, however, the total stress state at an arbitrary point is extremely complex, and its resolution can only be achieved through the mechanical analysis itself. In other words, aside from a few particularly simple problems—such as small-deflection pure bending of a one-dimensional beam—it is highly challenging to pre-estimate the stress state at a point in a deformable body prior to analysis. This situation makes it impossible to fix the elastic constants in the physical equations before the solution procedure. To overcome this difficulty, the simplified model presented in Figure 5 is introduced. By treating bending and mid-surface deformations as independent processes, this model allows the tensile and compressive stress states at each cross-sectional point to be predetermined. Through this approach, the definitive form of the physical equations is readily obtained, rendering the problem considered in this study solvable.

Because the simplified mechanical model proposed in this work treats the membrane stress and the bending stress at any point inside the shell as two independent components, different elastic moduli are assigned to the same material when the bending deformation and the mid-surface deformation are analyzed separately. For this reason, the model presented in Figure 5 possesses only approximate, partial physical consistency. Only when the membrane stress in the shell is dominant or the ratio *E*^+^/*E*^−^ approaches unity will this approximate treatment avoid introducing significant errors into the theoretical results. Meanwhile, classical shell stability theory [57] shows that the membrane stress of axially compressed thin cylindrical shells only on axial direction is indeed dominant during buckling. Thus, the simplified model is fully applicable to the problem under investigation. Furthermore, this model has already been applied in our earlier studies and its effectiveness has been validated by corresponding numerical simulations [45,46], which indirectly demonstrates the rationality of constructing the simplified model in the present work.

In summary, when mid-surface deformation alone is considered, the axial membrane stress in the shell under compressive loading can be directly identified as compressive. The circumferential membrane stress, by contrast, cannot be immediately determined, as discussed in the Introduction. A more detailed analysis of the deformation process of a thin cylindrical shell under uniform axial compression is therefore required. Because the existing literature provides no theoretical proof or discussion on this matter, the next subsection addresses this gap by analyzing how circumferential fibers in an axially compressed thin cylindrical shell deform before and after buckling, thereby supplying the theoretical basis for judging whether the circumferential membrane stress is tensile or compressive during buckling.

### 2.2. Determination of the Circumferential Membrane Stress State

From the discussion on the simplified mechanical model for bimodular materials in the previous subsection, it can be known that to analyze the buckling behavior of bimodular axially compressed thin cylindrical shells through theoretical methods, the initial determination of the tensile or compressive state of the circumferential membrane stress is of vital importance, and this subsection will conduct a detailed elaboration on this critical issue. Subsequently, the method for determining the state of the circumferential membrane stress in the shell under the linear buckling theory will be introduced.

For the linear buckling problem of axially compressed thin cylindrical shells under simply supported boundary conditions, the most typical buckling mode is that a certain number of sinusoidal half-waves form along the axial and circumferential directions of the shell, as shown in Figure 2. The deflection function *w*(*α*, *s*) that describes this deformation mode is given by Equation (1). According to the classical plate and shell theory, the circumferential membrane strain ${\bar{\varepsilon }}_{s,m}$ during the small deflection buckling of thin cylindrical shells can be expressed as:

(2) $${\bar{\varepsilon }}_{s,m}=\frac{\partial }{\partial s}v(\alpha ,s)-\frac{w(\alpha ,s)}{R},$$

where the subscript *m* stands for this strain component is the membrane strain, *v* is the displacement in the *s* direction, and it is a function of only two coordinate variables, *α* and *s*. By performing the circumferential closed integral with respect to the variable *s* on both sides of the above equation, the total deformation of mid-surface of the shell on circumferential direction Δ*l_c_* can be obtained as

(3) $$\Delta l_{c}=\int_{0}^{2\pi R}{\bar{\varepsilon }}_{s,m}ds=\int_{0}^{2\pi R}\frac{\partial }{\partial s}v(\alpha ,s)ds-\frac{1}{R}\int_{0}^{2\pi R}w(\alpha ,s)ds.$$

For cylindrical shell structures, to prevent the material from stacking or overlapping, the circumferential displacement should satisfy the closure condition, that is

(4) $$\int_{0}^{2\pi R}\frac{\partial }{\partial s}v(\alpha ,s)ds=0.$$

Substituting Equations (1) and (4) back into Equation (3), it can be found that the total deformation of the circumferential fibers Δ*l_c_* should satisfy

(5) $$\Delta l_{c}=-I_{s}\frac{f}{R}\sin\frac{m\pi \alpha }{L},$$

in which, the specific expression of *I_s_* is given by

(6) $$I_{s}=\frac{R}{n}\int_{0}^{2\pi n}\sin ydy,$$

here, *y* is the dimensionless parameter, and it satisfies *y* = *ns*/*R*. Obviously, since the circumferential buckling half-wave number *n* can only take positive integer values, the value of the definite integral *I_s_* is always zero. Combined with this conclusion, it can be inferred that, for thin cylindrical shells undergoing non-axisymmetric linear buckling under simply supported boundary conditions, the Δ*l_c_* is always equal to zero, that is,

(7) $$\Delta l_{c}=\int_{0}^{2\pi R}{\bar{\varepsilon }}_{s,m}ds\equiv 0.$$

This is consistent with the experimental phenomena observed by Kobayashi et al. [60], that is, when the axially compressed thin cylindrical shell structure undergoes buckling failure, the total deformation of mid-surface of the shell on circumferential direction on each of its cross-sections is almost equal to the original length, which enables the circumferential fibers of the shell to achieve non-tensile deformation. Equation (7) strictly proves the correctness of this conclusion, providing corresponding theoretical support for the above experimental phenomena. Next, by combined with the experimental phenomena observed by Kobayashi et al. [60] or the conclusion obtained from Equation (7), the circumferential membrane stress state of the shell can be determined.

From the introduction to the deformation characteristics of the shell in Section 2.1, it can be known that, since the mid-surface of the shell structure has an initial curvature, the membrane stress generated solely by the neutral surface deformation of the shell must also be considered in the small deflection deformation theory of the shell. However, due to the introduction of the bimodular characteristic of materials, when solving the membrane stresses in the shell through the equilibrium equations, the values of the elastic modulus and Poisson’s ratio in the equations will depend on the tensile or compressive stress state at that material point. To determine the values of the material constants in the equilibrium equations, we need to pre-judge only the tensile or compressive stress state of the membrane stresses in the axial and circumferential directions of the shell based on the deformation and mechanical characteristics of the shell before and after buckling, prior to solving the equations. It is worth noting that when only the mid-surface deformation of the shell is considered, the tensile or compressive stress state of the axial membrane stress induced by the uniform axial compressive load can be intuitively determined as the compressive stress state, while the tensile or compressive stress state of the circumferential membrane stress cannot be intuitively judged before the solution process. For this reason, we divide the entire process of the shell from the initial loading to the occurrence of buckling instability into two stages to analyze the change law of the internal circumferential membrane stress of the shell separately. The specific analysis conclusions are as follows:

Phase I: We take the process of gradually increasing the axial load on the shell from zero to before the occurrence of linear buckling failure as the first stage of the analysis. For the linear buckling theory, since the bending deformation before buckling is not considered, it can be considered that, at the moment immediately before buckling, the length of the circumferential fibers of the cylindrical shell is always equal to the original perimeter 2*πR*. However, due to the existence of the Poisson effect, the cylindrical shell will have a tendency of circumferential expansion during the process of axial compression along the *α* direction. It is worth noting that the simply supported boundary conditions at both ends of the shell shown in Figure 4 both constrain the displacement along the *s*-axis. Therefore, when only the mid-surface deformation is considered, the constraint along the circumferential direction brought by these simply supported boundary conditions will cause the circumferential fibers of the shells to be always under compression before buckling occurred. In addition, the circumferential deformation of the shell before buckling is extremely slight, so the membrane forces induced by such mid-surface deformation at this stage are neglected within the classical plate and shell stability theory, and this treatment is undoubtedly reasonable. Nevertheless, for the analysis carried out in the present work, accounting for the tensile or compressive state of membrane forces at this stage facilitates the formulation of physical and equilibrium equations, which renders detailed consideration and analysis necessary.

Phase II: On the basis of the first stage, we continue to increase the axial load until the shell undergoes linear buckling failure, and this stage is taken as the second stage of the analysis. According to the experimental phenomena observed by Kobayashi et al. [60]. (non-tensile deformation of the circumferential fibers) or Equation (7), it can be known that, after the shell undergoes linear buckling, the total length of its circumferential fibers remains unchanged, and the circumferential membrane stress of the shell has already been compressive before linear buckling occurs. Therefore, we can determine in advance that the circumferential membrane stress of the shell is always compressive before and after linear buckling.

In summary, we can draw the conclusion that the circumferential membrane stress, in the same way as the axial membrane stress, stays in the compressive state both prior to and after the occurrence of linear buckling. This conclusion will greatly simplify the construction of the physical equations for thin cylindrical shells under axial compression that take the bimodular effect into account.

## 3. Basic Equations of Bimodular Thin Cylindrical Shells

### 3.1. Geometric Equation

**Figure 6:**
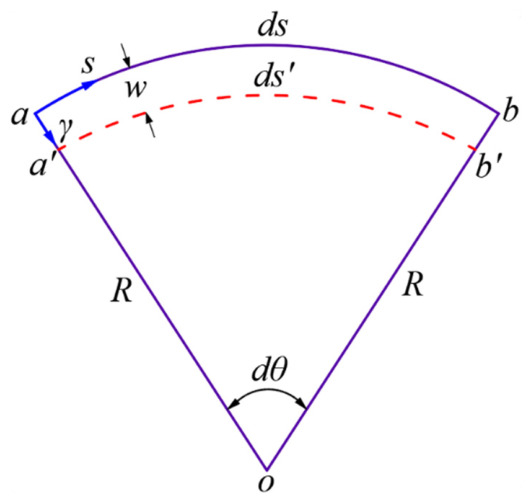
Mid-surface strain components due to circumferential deflection *w.*

For the mid-surface strain of axially compressed thin cylindrical shells under the small deformation assumption, the geometric nonlinear terms caused by the flexural deformation will not be considered. It is assumed that the in-plane displacements of the shell along the *α* and *s* directions are *u* and *v*, then the mid-surface strain caused by this displacement can be expressed as

(8) $$\varepsilon_{\alpha ,m}=\frac{\partial u}{\partial \alpha },\;\varepsilon_{s,m}=\frac{\partial v}{\partial s},\;{ϒ}_{\alpha s,m}=\frac{\partial v}{\partial \alpha }+\frac{\partial u}{\partial s},$$

the subscript *m* in above equation indicates that this strain is caused by the mid-surface deformation. However, since the cylindrical shell structure has an initial curvature 1/*R* along its circumferential direction, the change of the shell deflection will cause strain along the circumferential direction. As shown in Figure 6, it is assumed that the distance that the original arc length *ab* translates towards the center of the circle is *w*, the new arc length after translation is *a′b′*, then the strain of the arc length *ab* can be expressed as

(9) $${\varepsilon^{\prime }}_{s,m}=\frac{{ds}^{\prime }-ds}{ds}=\frac{(R-w)d\theta -Rd\theta }{Rd\theta }=-\frac{w}{R}.$$

Combining Equations (8) and (9), the total mid-surface strain of the shell under the small deformation assumption can be obtained as

(10) $$\left\{\begin{array}{c} {\bar{\varepsilon }}_{\alpha ,m}=\varepsilon_{\alpha ,m}=\frac{\partial u}{\partial \alpha }\\ {\bar{\varepsilon }}_{s,m}=\varepsilon_{s,m}+{\varepsilon^{\prime }}_{s,m}=\frac{\partial v}{\partial s}-\frac{w}{R}\\ {\bar{ϒ}}_{\alpha s,m}={ϒ}_{\alpha s,m}=\frac{\partial v}{\partial \alpha }+\frac{\partial u}{\partial s} \end{array}\right..$$

Meanwhile, considering that Equation (10) describes the in-plane deformation of the shell, it can also be referred to as the in-plane geometric equation.

Moreover, the bending strain induced by cross-sectional torsion may be expressed as

(11) $$\varepsilon_{\alpha ,b}=-\chi_{\alpha }\gamma ,\;\varepsilon_{s,b}=-\chi_{s}\gamma ,\;{ϒ}_{\alpha s,b}=-2\chi_{\alpha s}\gamma ,$$

where the subscript *b* identifies this strain component as the bending strain; *χ_a_*, *χ_s_*, and *χ_as_* respectively represent the curvature and twist rate along the associated directions, which are defined as

(12) $$\chi_{\alpha }=\frac{\partial^{2}w}{\partial \alpha^{2}},\;\chi_{s}=\frac{\partial^{2}w}{\partial s^{2}},\chi_{\alpha s}=\frac{\partial^{2}w}{\partial \alpha \partial s}.$$

Equations (11) and (12) together constitute the out-of-plane geometrical equation.

In summary, the total strains of the shell along the *α*, *s*, *γ* directions *e_α_*, *e_s_*, *e_γ_* can be expressed as

(13) $$e_{\alpha }={\bar{\varepsilon }}_{\alpha }-\chi_{\alpha }\gamma ,\;e_{s}={\bar{\varepsilon }}_{s}-\chi_{s}\gamma ,e_{\alpha s}={\bar{\varepsilon }}_{\alpha s}-2\chi_{\alpha s}\gamma .$$

### 3.2. Physical Equation

Based on the stress-state analysis given in Section 2.1, the total stress on the cross-section of an axially compressed thin cylindrical shell that exhibits the bimodular effect may be regarded as the sum of two separate contributions: the bending stress and the membrane stress. For this reason, the present section will develop separate physical equations to describe these two types of stress, respectively. To begin with, when considering the membrane stress component, the circumferential membrane stress state we have determined in Section 2.2 reveals that the membrane stresses along both the circumferential and axial directions remain in the compressive state across the entire thickness of the cross-section. Accordingly, the physical equations corresponding to this part of the analysis can be formulated as follows

(14) $$\left\{\begin{array}{l} \sigma_{\alpha ,m}^{-}=\frac{E^{-}}{1-{\left(\mu^{-}\right)}^{2}}({\bar{\varepsilon }}_{\alpha ,m}+\mu^{-}{\bar{\varepsilon }}_{s,m})\\ \sigma_{s,m}^{-}=\frac{E^{-}}{1-{\left(\mu^{-}\right)}^{2}}({\bar{\varepsilon }}_{s,m}+\mu^{-}{\bar{\varepsilon }}_{\alpha ,m})\\ \tau_{\alpha s,m}^{-}=\frac{E^{-}}{2(1+\mu^{-})}{\bar{ϒ}}_{\alpha s,m} \end{array}\right.,$$

where *σ_i_* and *τ_i_* designate the normal and shear stresses, while *ε_i_* and ϒ*_i_* designate the corresponding normal and shear strains. Since the membrane stress is uniformly distributed through the cross-sectional thickness, the resulting internal forces are given by

(15) $$\left\{\begin{array}{c} F_{\alpha ,m}=\frac{E^{-}t}{1-{\left(\mu^{-}\right)}^{2}}({\bar{\varepsilon }}_{\alpha ,m}+\mu^{-}{\bar{\varepsilon }}_{s,m})\\ \begin{array}{l} F_{s,m}=\frac{E^{-}t}{1-{\left(\mu^{-}\right)}^{2}}({\bar{\varepsilon }}_{s,m}+\mu^{-}{\bar{\varepsilon }}_{\alpha ,m})\\ F_{\alpha s,m}=\frac{E^{-}t}{2(1+\mu^{-})}{\bar{ϒ}}_{\alpha s,m} \end{array} \end{array}\right..$$

where *F_α_*_,*m*_, *F_s_*_,*m*_, and *F_αs_*_,*m*_ are the resultants of membrane stress.

For the bending stress, the corresponding bending strain is shown in Equation (11). Meanwhile, considering the bimodular effect of the material, when deriving the physical equations related to the bending deformation, it is necessary to classify the bending stress of the tensile and compressive subarea according to the simplified mechanical model shown in Figure 5a. For the tensile region of the structure (0 ≤ *γ* ≤ *t*_1_),

(16a) $$\left\{\begin{array}{c} \sigma_{\alpha ,b}^{+}=\frac{E^{+}}{1-{\left(\mu^{+}\right)}^{2}}(\varepsilon_{\alpha ,b}+\mu^{+}\varepsilon_{s,b})=-\frac{E^{+}\gamma }{1-{\left(\mu^{+}\right)}^{2}}(\chi_{\alpha }+\mu^{+}\chi_{s})\\ \begin{array}{l} \sigma_{s,b}^{+}=\frac{E^{+}}{1-{\left(\mu^{+}\right)}^{2}}(\varepsilon_{s,b}+\mu^{+}\varepsilon_{\alpha ,b})=-\frac{E^{+}\gamma }{1-{\left(\mu^{+}\right)}^{2}}(\chi_{s}+\mu^{+}\chi_{\alpha })\\ \tau_{\alpha s,b}^{+}=\frac{E^{+}}{2(1+\mu^{+})}{ϒ}_{\alpha s,b}=-\frac{E^{+}\gamma }{\left(1+\mu^{+}\right)}\chi_{\alpha s} \end{array} \end{array}\right.,$$

and for the compressive region of the shell (−*t*_2_ ≤ *γ* ≤ 0),

(16b) $$\left\{\begin{array}{c} \sigma_{\alpha ,b}^{-}=\frac{E^{-}}{1-{\left(\mu^{-}\right)}^{2}}(\varepsilon_{\alpha ,b}+\mu^{-}\varepsilon_{s,b})=-\frac{E^{-}\gamma }{1-{\left(\mu^{-}\right)}^{2}}(\chi_{\alpha }+\mu^{-}\chi_{s})\\ \begin{array}{l} \sigma_{s,b}^{-}=\frac{E^{-}}{1-{\left(\mu^{-}\right)}^{2}}(\varepsilon_{s,b}+\mu^{-}\varepsilon_{\alpha ,b})=-\frac{E^{-}\gamma }{1-{\left(\mu^{-}\right)}^{2}}(\chi_{s}+\mu^{-}\chi_{\alpha })\\ \tau_{\alpha s,b}^{-}=\frac{E^{-}}{2(1+\mu^{-})}{ϒ}_{\alpha s,b}=-\frac{E^{-}\gamma }{\left(1+\mu^{-}\right)}\chi_{\alpha s} \end{array} \end{array}\right..$$

According to the definitions of bending moment and torque, by integrating Equation (16) along the *γ* axis according to the corresponding tensile and compressive subarea, it can be obtained that

(17) $$\left\{\begin{array}{c} M_{\alpha }=-\frac{E^{+}t_{1}^{3}}{3[1-{\left(\mu^{+}\right)}^{2}]}(\chi_{\alpha }+\mu^{+}\chi_{s})-\frac{E^{-}t_{2}^{3}}{3[1-{\left(\mu^{-}\right)}^{2}]}(\chi_{\alpha }+\mu^{-}\chi_{s})\\ \begin{array}{l} M_{s}=-\frac{E^{+}t_{1}^{3}}{3[1-{\left(\mu^{+}\right)}^{2}]}(\chi_{s}+\mu^{+}\chi_{\alpha })-\frac{E^{-}t_{2}^{3}}{3[1-{\left(\mu^{-}\right)}^{2}]}(\chi_{s}+\mu^{-}\chi_{\alpha })\\ M_{\alpha s}=-\frac{1}{3}\left[\frac{E^{+}t_{1}^{3}}{\left(1+\mu^{+}\right)}+\frac{E^{-}t_{2}^{3}}{\left(1+\mu^{-}\right)}\right]\chi_{\alpha s} \end{array} \end{array}\right.,$$

where *M_α_*, *M_s_*, and *M_αs_* are the bending moment and torque. If we let

(18) $$D^{+}=\frac{E^{+}t_{1}^{3}}{3[1-{\left(\mu^{+}\right)}^{2}]},D^{-}=\frac{E^{-}t_{2}^{3}}{3[1-{\left(\mu^{-}\right)}^{2}]},$$

which satisfies

(19) $$D^{*}=D^{+}+D^{-},$$

where D^*^ is the equivalent bending stiffness of the bimodular shell introduced to facilitate subsequent derivations and simplify the expressions of equilibrium equations. Since Equation (18) is obtained by integrating over different regions on the same cross-section of the shell, the bending stiffness of the bimodular shell over the entire cross-section can be directly obtained by superposing the two terms in Equation (18). Meanwhile, with the aid of Equation (18), Equation (17) can be rewritten as

(20) $$\left\{\begin{array}{c} M_{\alpha }=-D^{+}(\chi_{\alpha }+\mu^{+}\chi_{s})-D^{-}(\chi_{\alpha }+\mu^{-}\chi_{s})\\ \begin{array}{l} M_{s}=-D^{+}(\chi_{s}+\mu^{+}\chi_{\alpha })-D^{-}(\chi_{s}+\mu^{-}\chi_{\alpha })\\ M_{\alpha s}=-[(1-\mu^{+})D^{+}+(1-\mu^{-})D^{-}]\chi_{\alpha s} \end{array} \end{array}\right..$$

Equations (15) and (17) are the internal force equations associated with membrane forces, bending moments and torque. Furthermore, given the selection characteristic of the coordinate system origin in the simplified mechanical model of this work, the bending deformation and the mid-surface deformation are uncoupled with each other. Therefore, when solely the bending deformation of the shell is taken into account, the normal resultant forces on the cross-sections along the two principal curvature directions must fulfill *N_α_* = *N_s_* = 0. Together with Equation (16), this condition yields

(21a) $$N_{\alpha }=\int_{0}^{t_{1}}\sigma_{\alpha ,b}^{+}d\gamma +\int_{-t_{2}}^{0}\sigma_{\alpha ,b}^{-}d\gamma =-\frac{E^{+}t_{1}^{2}}{2[1-{\left(\mu^{+}\right)}^{2}]}(\chi_{\alpha }+\mu^{+}\chi_{s})+\frac{E^{-}t_{2}^{2}}{2[1-{\left(\mu^{-}\right)}^{2}]}(\chi_{\alpha }+\mu^{-}\chi_{s})=0,$$

and

(21b) $$N_{s}=\int_{0}^{t_{1}}\sigma_{s,b}^{+}d\gamma +\int_{-t_{2}}^{0}\sigma_{s,b}^{-}d\gamma =-\frac{E^{+}t_{1}^{2}}{2[1-{\left(\mu^{+}\right)}^{2}]}(\chi_{s}+\mu^{+}\chi_{\alpha })+\frac{E^{-}t_{2}^{2}}{2[1-{\left(\mu^{-}\right)}^{2}]}(\chi_{s}+\mu^{-}\chi_{\alpha })=0.$$

Adding Equation (21a,b) will yield

(22) $$\frac{E^{+}t_{1}^{2}}{1-\mu^{+}}=\frac{E^{-}t_{2}^{2}}{1-\mu^{-}},$$

combining above equation with *t*_1_ + *t*_2_ = *t*, the neutral surface position can be determined by the formula as follows

(23) $$\frac{t_{1}}{t}=\frac{\sqrt{E^{-}(1-\mu^{+})}}{\sqrt{E^{+}(1-\mu^{-})}+\sqrt{E^{-}(1-\mu^{+})}},\frac{t_{2}}{t}=\frac{\sqrt{E^{+}(1-\mu^{-})}}{\sqrt{E^{+}(1-\mu^{-})}+\sqrt{E^{-}(1-\mu^{+})}},$$

where *t*_1_ and *t*_2_ are the tensile zone thickness and compressive zone thickness, respectively.

**Figure 7:**
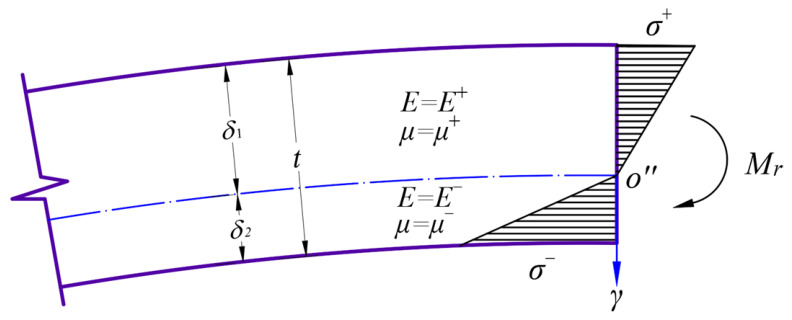
Tension-compression zones under reversed bending moments *Mr* in the outward convex region of the shell.

It is worth noting that Equation (23) indirectly determines the position of the neutral layer by obtaining the respective thicknesses of the tensile and compressive regions, and it does not directly represent the specific coordinate of the neutral layer on the cross-section of the shell. That is, the final position of the neutral layer can only be obtained by determining the bending direction of the shell. Since the internal force Equation (17) were derived based on the direction of bending moment shown in Figure 5a, it can be seen from the schematic diagram of the non-axisymmetric buckling mode of the simply supported axially compressed thin cylindrical shell presented in Figure 2 that Equation (17) represents the physical equations for the regions of the shell exhibiting an inward concave deformation tendency. However, it can be seen from Figure 2 that in addition to the inward concave regions, a considerable portion of the shell regions exhibits an outward convex deformation tendency. Therefore, for the outward convex regions, the physical equations corresponding to the bending moment with the opposite direction to that in Figure 5a need to be considered. When the bending moment in Figure 5a is reversed (i.e., the cross-section is located in the outward convex region), the position of its physical neutral layer will change accordingly. Nevertheless, since the simplified mechanical model of this work always places the coordinate origin *o*” on the neutral layer, the bending stress distribution in the tensile and compressive regions under the reversed bending moment can still be described by Equation (16). It is assumed that the thickness of the tensile region under the reversed bending moment *Mr* is denoted by *δ*_1_, and that of the compressive region is denoted by *δ*_2_, and the corresponding subarea model is shown in Figure 7. Similarly, by the aforementioned method of setting the normal resultant force to zero, we can solve that

(24a) $$N_{\alpha }=\int_{-\delta_{1}}^{0}\sigma_{\alpha ,b}^{+}d\gamma +\int_{0}^{\delta_{2}}\sigma_{\alpha ,b}^{-}d\gamma =\frac{E^{+}\delta_{1}^{2}}{2[1-{\left(\mu^{+}\right)}^{2}]}(\chi_{\alpha }+\mu^{+}\chi_{s})-\frac{E^{-}\delta_{2}^{2}}{2[1-{\left(\mu^{-}\right)}^{2}]}(\chi_{\alpha }+\mu^{-}\chi_{s})=0,$$

and

(24b) $$N_{s}=\int_{-\delta_{1}}^{0}\sigma_{s,b}^{+}d\gamma +\int_{0}^{\delta_{2}}\sigma_{s,b}^{-}d\gamma =\frac{E^{+}\delta_{1}^{2}}{2[1-{\left(\mu^{+}\right)}^{2}]}(\chi_{s}+\mu^{+}\chi_{\alpha })-\frac{E^{-}\delta_{2}^{2}}{2[1-{\left(\mu^{-}\right)}^{2}]}(\chi_{s}+\mu^{-}\chi_{\alpha })=0.$$

Similarly, from Equation (24) and *δ*_1_ + *δ*_2_ = *t*, we can obtain the respective thicknesses of the tensile and compressive regions under the reversed bending moment as

(25) $$\frac{\delta_{1}}{t}=\frac{\sqrt{E^{-}(1-\mu^{+})}}{\sqrt{E^{+}(1-\mu^{-})}+\sqrt{E^{-}(1-\mu^{+})}},\frac{\delta_{2}}{t}=\frac{\sqrt{E^{+}(1-\mu^{-})}}{\sqrt{E^{+}(1-\mu^{-})}+\sqrt{E^{-}(1-\mu^{+})}}.$$

Clearly, the above equation indicates that *δ*_1_ and *δ*_2_ are numerically identical to *t*_1_ and *t*_2_ in Equation (23), with the only difference being the positions of the tensile and compressive regions on the cross-section. Given that *δ*_1_ ≠ *δ*_2_, the shift of the tensile-compressive region positions will in turn cause the shift of the neutral layer.

Furthermore, by integrating Equation (16) over the region shown in Figure 7, we can obtain the expression of the bending stiffness under the reversed bending moment as

(26) $$D_{r}^{*}=D_{r}^{+}+D_{r}^{-}=\frac{E^{+}\delta_{1}^{3}}{3[1-{\left(\mu^{+}\right)}^{2}]}+\frac{E^{-}\delta_{2}^{3}}{3[1-{\left(\mu^{-}\right)}^{2}]}.$$

since *δ*_1_ = *t*_1_ and *δ*_2_ = *t*_2_, the bending stiffness under the reversed bending moment D^*^_r_ is also numerically identical to D^*^. That is, under the reversed bending moment, although the position of the neutral layer of the shell shifts, its internal force equations can still be described by Equation (20). Therefore, the subsequent derivation of the governing equations will no longer distinguish or discuss the influence of the shell’s bending direction.

## 4. Theoretical Solution of the Linear Critical Load

### 4.1. Derivation of the Governing Equations

Based on the general shell stability theory [61], the differential equations of equilibrium for the buckling problem of axially compressed thin cylindrical shells can be approximately derived from the bending theory of constant-curvature shallow shells. When the constant-curvature shallow shells undergo small deflection bending under transverse loads, the equilibrium equations and compatibility equations are, respectively

(27) $$\left\{\begin{array}{l} \frac{\partial F_{\alpha ,m}}{\partial \alpha }+\frac{\partial F_{s\alpha ,m}}{\partial s}=0,\;\frac{\partial F_{s,m}}{\partial s}+\frac{\partial F_{\alpha s,m}}{\partial \alpha }=0\;\\ \frac{\partial M_{\alpha }}{\partial \alpha }+\frac{\partial M_{s\alpha }}{\partial s}-Q_{\alpha }=0,\;\frac{\partial M_{s}}{\partial s}+\frac{\partial M_{\alpha s}}{\partial \alpha }-Q_{s}=0\;\\ \frac{\partial Q_{\alpha }}{\partial \alpha }+\frac{\partial Q_{s}}{\partial s}+F_{\alpha ,m}(\chi_{\alpha }+k_{\alpha })+2F_{s\alpha ,m}\chi_{\alpha s}+F_{s,m}(\chi_{s}+k_{s})+q_{L}=0 \end{array}\right.,$$

and

(28) $$\frac{\partial^{2}{\bar{\varepsilon }}_{s,m}}{\partial \alpha^{2}}+\frac{\partial^{2}{\bar{\varepsilon }}_{\alpha ,m}}{\partial s^{2}}-\frac{\partial^{2}{\bar{ϒ}}_{\alpha s,m}}{\partial \alpha \partial s}=-\frac{1}{R}\chi_{\alpha },$$

where *Q_α_* and *Q_s_* are the lateral shear forces per unit width, and *q_L_* is the lateral load; *k_α_* and *k_s_* represent the initial curvatures of the constant-curvature shallow shells along the *α* and *s* axes, respectively. For cylindrical shell structures, their initial curvature can be easily determined as

(29) $$k_{\alpha }=0,k_{s}=\frac{1}{R}.$$

To fulfill the first two relations of Equation (27), the membrane forces may be represented by the Airy stress function Φ(α, s) as

(30) $$F_{\alpha ,m}=\frac{\partial^{2}\Phi }{\partial s^{2}},F_{s,m}=\frac{\partial^{2}\Phi }{\partial \alpha^{2}},F_{\alpha s,m}=-\frac{\partial^{2}\Phi }{\partial \alpha \partial s}.$$

Because the thin cylindrical shell now bears only an axial compressive load with no lateral component, the term *q_L_* in Equation (27) is set to zero. By combining with the last three expressions of Equation (27), and substituting Equations (12), (20) and (29) into them, we obtain

(31) $$D^{*}\nabla^{4}w=F_{\alpha ,m}\frac{\partial^{2}w}{\partial \alpha^{2}}+2F_{s\alpha ,m}\frac{\partial^{2}w}{\partial \alpha \partial s}+F_{s,m}\left(\frac{\partial^{2}w}{\partial s^{2}}+\frac{1}{R}\right),$$

where $\nabla^{4}$ is the biharmonic operator, which is defined as

(32) $$\nabla^{4}=\frac{\partial^{4}}{\partial \alpha^{4}}+2\frac{\partial^{4}}{\partial \alpha^{2}\partial s^{2}}+\frac{\partial^{4}}{\partial s^{4}}.$$

In addition, substituting Equations (12), (15), (29) and (30) into Equation (28) yields

(33) $$\frac{1}{E^{-}t}\nabla^{4}\Phi =-\frac{1}{R}\frac{\partial^{2}w}{\partial \alpha^{2}}.$$

### 4.2. Derivation of the Linear Critical Load

Through the above derivation, we have obtained the linear buckling governing equations of the bimodular axially compressed thin cylindrical shells, that is, Equations (31) and (33). According to the classical shell stability theory, to obtain the analytical expression of its linear critical load, it is also necessary to rewrite the above governing equations into a coupled form. Now, multiplying both sides of Equation (31) by $\nabla^{4}$, and substituting into Equation (33), we can obtain a linear buckling governing equation in coupled form as

(34) $$D^{*}\nabla^{4}\nabla^{4}w+\frac{E^{-}t}{R^{2}}\frac{\partial^{4}w}{\partial \alpha^{4}}=F_{\alpha ,m}\frac{\partial^{2}(\nabla^{4}w)}{\partial \alpha^{2}}+2F_{\alpha s,m}\frac{\partial^{2}(\nabla^{4}w)}{\partial \alpha \partial s}+F_{s,m}\frac{\partial^{2}(\nabla^{4}w)}{\partial s^{2}},$$

in which, the membrane forces on the right-hand side are only related to the initial loading state of the shell. Since the shell is merely subjected to axial compressive force at this stage, the external forces *F_s_*_,*m*_ and *F_αs_*_,*m*_ along the circumferential direction of the shell are both equal to zero. Let the magnitude of the uniform axial compressive force acting on the shell be *F_αs_*_,*m*_ = −*q_α_t*, then the above equation can be further simplified as

(35) $$D^{*}\nabla^{4}\nabla^{4}w+\frac{E^{-}t}{R^{2}}\frac{\partial^{4}w}{\partial \alpha^{4}}+q_{\alpha }t\frac{\partial^{2}(\nabla^{4}w)}{\partial \alpha^{2}}=0,$$

which is exactly the linear buckling governing equation in coupled form for bimodular axially compressed thin cylindrical shells. By substituting the specific expression of the deflection function *w*(*α*, *s*), the corresponding linear critical buckling load can be obtained. Substituting the deflection function shown in Equation (1) into Equation (35), we obtain

(36) $$\left[D^{*}{\left(\frac{m^{2}\pi^{2}}{L^{2}}+\frac{n^{2}}{R^{2}}\right)}^{4}+\frac{E^{-}tm^{4}\pi^{4}}{R^{2}L^{4}}-\frac{q_{\alpha }tm^{2}\pi^{2}}{L^{2}}{\left(\frac{m^{2}\pi^{2}}{L^{2}}+\frac{n^{2}}{R^{2}}\right)}^{2}\right]f\sin\left(\frac{m\pi \alpha }{L}\right)\sin\left(\frac{ns}{R}\right)=0.$$

It is worth noting that the deflection function in the above equation *w* = *f*sin(*mπα/L*)sin(*ns*/*R*) has a non-vanishing amplitude when the shell undergoes buckling. Therefore, we can only set the coefficient of the deflection function in Equation (36) to be identically zero, that is,

(37) $$D^{*}{\left(\frac{m^{2}\pi^{2}}{L^{2}}+\frac{n^{2}}{R^{2}}\right)}^{4}+\frac{E^{-}tm^{4}\pi^{4}}{R^{2}L^{4}}-\frac{q_{\alpha }tm^{2}\pi^{2}}{L^{2}}{\left(\frac{m^{2}\pi^{2}}{L^{2}}+\frac{n^{2}}{R^{2}}\right)}^{2}=0,$$

from which, the axial compressive load *q_α_* of the shell can be expressed as

(38) $$q_{\alpha }=\frac{D^{*}}{t}\frac{L^{2}}{m^{2}\pi^{2}}{\left(\frac{m^{2}\pi^{2}}{L^{2}}+\frac{n^{2}}{R^{2}}\right)}^{2}+\frac{E^{-}}{R^{2}}\frac{m^{2}\pi^{2}}{L^{2}}{\left(\frac{m^{2}\pi^{2}}{L^{2}}+\frac{n^{2}}{R^{2}}\right)}^{-2}.$$

To evaluate the critical load, we introduce the following inequality

(39) $${\left(\sqrt{a}X-\sqrt{b}\frac{1}{X}\right)}^{2}\geq 0,$$

in which, *a* and *b* are arbitrary positive real numbers, and *X* is the variable. Clearly, this equation holds identically for any real number *X*. Meanwhile, by expanding the above equation into a perfect square, we can further obtain the following inequality

(40) $$aX^{2}+b\frac{1}{X^{2}}\geq 2\sqrt{ab}.$$

By combining Equations (38) and (40), we obtain

(41) $$q_{\alpha }\geq \frac{2}{tR}\sqrt{D^{*}E^{-}t}.$$

From the above equation, we can derive the minimum value *q*_min_ of the shell’s axial compressive load *q_α_*, which is exactly the linear critical load *q_cr_* of the bimodular axially compressed thin cylindrical shells

(42) $$q_{cr}=q_{\min}=\frac{2}{tR}\sqrt{D^{*}E^{-}t}=\frac{2t}{R}\sqrt{\frac{E^{-}}{3}\left[\frac{E^{+}}{1-{\left(\mu^{+}\right)}^{2}}{\left(\frac{t_{1}}{t}\right)}^{3}+\frac{E^{-}}{1-{\left(\mu^{-}\right)}^{2}}{\left(\frac{t_{2}}{t}\right)}^{3}\right]}.$$

## 5. Examples and Numerical Simulation

In this section, we conduct numerical simulations to verify the validity of the simplified mechanical model and the theoretical solution proposed in this work. Furthermore, we compare the results with those of previous researchers to further demonstrate the advantages of the theoretical solution in this study.

### 5.1. Computational Examples

This study employs the material parameters of aluminum alloy 6061-T6 (AA 6061-T6) as the reference for numerical simulation and computation. The basic mechanical properties of AA 6061-T6 are: density = 2700 g/mm^3^, elastic modulus *E* = 68.9 GPa, and Poisson’s ratio *μ* = 0.33.

To facilitate the subsequent analysis, the mean values of the elastic modulus and Poisson’s ratio are kept constant when the bimodular characteristic is taken into account; i.e., *E* = (*E*^+^ + *E*^−^)/2 and *μ* = (*μ*^+^ + *μ*^−^)/2 are held fixed. Moreover, to retain the symmetry of the material Jacobian matrix, the tensile and compressive elastic moduli and Poisson’s ratios are typically assumed to satisfy *μ*^+^/*E*^+^ = *μ*^−^/*E*^−^. Without loss of generality, we consider five different combinations of *E*^+^/*E*^−^, which are sequentially 1/2, 2/3, 1, 3/2, and 2. Under the above conditions, all the specific values of *E*^+^, *E*^−^, *μ*^+^, and *μ*^−^ corresponding to these five combinations can be calculated individually, as listed in Table 1.

Meanwhile, to facilitate the subsequent parametric analysis, we select several groups of cylindrical shells with different geometric dimensions. Specifically, the radius *R* takes values of 200 mm and 250 mm, the shell height *L* takes values of 200 mm, 300 mm, and 400 mm, and the shell thickness *t* takes values of 1 mm and 2 mm. Among the above values, the minimum radius-to-thickness ratio *R/t* of the shells is 100, which satisfies the basic definition of thin shells (*R/t* > 20). The specific combinations of geometric parameters are listed in Table 2. In addition, in the buckling analysis of cylindrical shells, the Batdorf parameter *Z* = (1−*μ*^2^)^1/2^(*L*^2^/*Rt*) is usually adopted to characterize the geometric properties of the shells. A smaller value of *Z* indicates a shorter, squatter shell, while a larger value indicates a taller, slenderer one. To eliminate the influence caused by the variation of Poisson’s ratio, in this work we adopt $\bar{Z}$= *L*^2^/*Rt* to replace *Z*, and the value of $\bar{Z}$ is also provided in Table 2.

Based on the data in Table 1 and Table 2, we can obtain the linear critical loads of the bimodular thin cylindrical shells under axial compressive load, which can be further compared with the numerical simulation results. Furthermore, as early as more than 50 years ago, Jones [62] has investigated the small-deflection biaxial buckling behavior of bimodular thin cylindrical shells under combined axial compression and external pressure. Nevertheless, different from the simplified mechanical model proposed in this work, Jones strictly adopted the original bimodular material constitutive relation proposed by Ambartsumyan, i.e., the tensile or compressive stress state at a point in the material is determined by the sign of the principal stress at that point, and based on this, he derived the calculation formula for the critical load *q_x_^cr^* of bimodular thin cylindrical shells under pure axial compression as follows

(43) $$q_{x}^{cr}=\frac{t}{R}\frac{E^{-}}{1-{\left(\mu^{-}\right)}^{2}E^{+}/E^{-}}\sqrt{\frac{E^{+}(1-\mu^{+}\mu^{-})}{3E^{-}(1+\sqrt{E^{+}/E^{-}})}\left[1+\frac{\left(1+\mu^{+}\right)}{\left(1+\mu^{-}\right)}\sqrt{\frac{E^{-}}{E^{+}}}\right]}.$$

With the above equation, we will, in the next subsection, compare it with the theoretical solution of this work and the numerical simulation results together in the same numerical example.

It is worth noting that Jones’ solution does not fully account for the influence of the bimodular effect of materials on the bending stiffness of the shell. Specifically, when deriving Equation (43), Jones indiscriminately assigned the elastic constants in the bending stiffness as either the tensile or compressive elastic modulus over the entire cross-section of the shell based on the sign of the principal stress. However, when considering the bending deformation of the shell cross-section, as shown in Figure 5a, the fibers on both sides of the physical neutral layer do not elongate or shorten simultaneously. Consequently, the calculation of the lateral bending stiffness of the shell in Jones’ solution is insufficiently accurate and inconsistent with actual conditions. Furthermore, Jones’ solution assumes that the total circumferential stress of the shell after buckling is entirely tensile, which is clearly inconsistent with the actual situation. It is readily apparent from the schematic diagram of the shell buckling mode shown in Figure 2 that the circumferential fibers of the shell are actually in an alternating tension-compression stress state. Therefore, the consideration of the stress state of the circumferential fibers of the shell in Equation (43) is also insufficiently accurate and deviates from the actual stress state of the shell.

To address the deficiencies of Jones’ solution, the mechanical model proposed in this paper re-derives a critical load calculation formula that fully accounts for the influence of the bimodular effect of materials on the bending stiffness of the shell. Moreover, although the mechanical model in this paper concludes that the circumferential fibers in the mid-surface deformation part of the shell are always in a compressive stress state, when this stress state is superposed with that from the bending deformation part to obtain the total circumferential fiber stress state of the shell, the total circumferential fiber stress state will be in an alternating tension-compression state, which depends on the bending direction of the circumferential buckling half-waves of the shell. The implications of these theoretical improvements will be further elucidated through comparisons with a series of numerical examples in the subsequent sections.

### 5.2. Numerical Simulation

In this section, we conduct numerical simulations and compare the simulation results with the theoretical solution of this work as well as the theoretical results of Jones. In ABAQUS, the Eigen Value buckling analysis in the Standard module is commonly adopted as the simulation method for the linear buckling behavior of axially compressed thin cylindrical shells. Since the constitutive model of bimodular materials is not included in the material library of current commercial finite element software, for this purpose, based on our previous studies on this material [45], we developed a user material subroutine (UMAT) in ABAQUS/CAE 2021, which is applicable to the Eigen Value buckling analysis of axially compressed thin cylindrical shells.

When using the UMAT subroutine to conduct secondary development for ABAQUS, the first issue we need to consider is the constitutive model of the material. The existing numerical calculation models for bimodular materials are mainly established based on the sign criterion of principal stress proposed by Ambartsumyan [20]. For this purpose, in this work, we define the elastic modulus at a point in the material based on Ambartsumyan’s model for judging the principal stress state. The main calculation steps of this program are as follows

(i) At the first increment, we receive the material parameters from the ABAQUS main program, and respectively define the elastic moduli along the thickness direction of the bimodular material under tensile and compressive states.

(ii) Based on the classical Jacobi method, we can calculate the Eigen Values and eigenvectors of the stress matrix to obtain the principal stresses and principal directions.

(iii) According to the obtained principal stress directions, we can determine the corresponding constitutive relation of each element, and assemble the stiffness matrix of each element to form the global stiffness matrix. Its two-dimensional tensor form is as follows

(44) $$D=\left[\begin{array}{cccccc} a_{11} & a_{12} & a_{13} & 0 & 0 & 0\\ a_{21} & a_{22} & a_{23} & 0 & 0 & 0\\ a_{31} & a_{32} & a_{33} & 0 & 0 & 0\\ 0 & 0 & 0 & G & 0 & 0\\ 0 & 0 & 0 & 0 & G & 0\\ 0 & 0 & 0 & 0 & 0 & G \end{array}\right],$$

where *a_ij_* (*i*, *j* = 1, 2, 3) are the compliance coefficients at the current material integration point, and *G* is the shear modulus of the material. For bimodular materials, the compliance coefficients *a_ij_* (*i*, *j* = 1, 2, 3) can be defined as

(45) $$a_{ij}=\left\{\begin{array}{l} \frac{E_{i}}{-1+\mu_{1}\mu_{2}+\mu_{2}\mu_{3}+\mu_{1}\mu_{3}+2\mu_{1}\mu_{2}\mu_{3}}\left(-1+\frac{1}{\mu_{i}}\prod_{k=1}^{3}\mu_{k}\right)i=j\\ \frac{-E_{i}}{-1+\mu_{1}\mu_{2}+\mu_{2}\mu_{3}+\mu_{1}\mu_{3}+2\mu_{1}\mu_{2}\mu_{3}}\left(-\mu_{j}+\frac{1}{\mu_{i}}\prod_{k=1}^{3}\mu_{k}\right)i\neq j \end{array}\right.\;\;i,j=1,2,3,$$

where *E_i_* and *μ_i_* are the elastic modulus and Poisson’s ratio in the direction *i*, which are determined by the calculated principal stress directions; meanwhile, for bimodular materials, the value of the shear modulus *G* in Equation (44) is determined by the following calculation formula

(46) $$G=\frac{\eta E^{+}+\left(1-\eta \right)E^{-}}{2\eta \left(1+\mu^{+}\right)+2\left(1-\eta \right)\left(1+\mu^{-}\right)},$$

in which, *η* is the acceleration convergence factor, which determines the value of *G* by the weight of the sum of principal tensile stresses relative to the sum of the absolute values of principal stresses at the material integration point. For example, if the tensile-compressive state of the principal stresses at the current integration point satisfies *σ*_11_ > 0, *σ*_22_ < 0, *σ*_33_ < 0, then *η* can be written as

(47) $$\eta =\frac{\sigma_{11}}{\left|\sigma_{11}\right|+\left|\sigma_{22}\right|+\left|\sigma_{33}\right|}.$$

Obviously, the value of *η* satisfies 0 ≤ *η* ≤ 1.

(iv) After obtaining the Jacobian matrix of the bimodular material, the next step is to update the stress state at the current moment. In the ABAQUS/Standard module, the objective rate adopted for the stress update of solid elements is the Jaumann rate; therefore, we only need to implement one step in the UMAT subroutine, that is

(48) $$\sigma_{t+\Delta t}=\Delta R\cdot \sigma_{t}\cdot \Delta R^{T}+\Delta \sigma ,$$

where *σ_t_*_+Δ_*_t_* is the updated stress matrix within the time increment Δ*t*. The co-axial rotation of the stress at time *t*, i.e., Δ*R*·*σ_t_*·Δ*R^T^*, has been implemented in the ABAQUS program; therefore, the UMAT only needs to calculate the stress increment Δ*σ* at time *t* to update the stress state. If the equilibrium condition is satisfied based on the stress matrix *σ_t_*_+Δ_*_t_*, the next increment step will be executed; otherwise, the size of the iteration step will be readjusted until the equilibrium condition is met.

**Figure 8:**
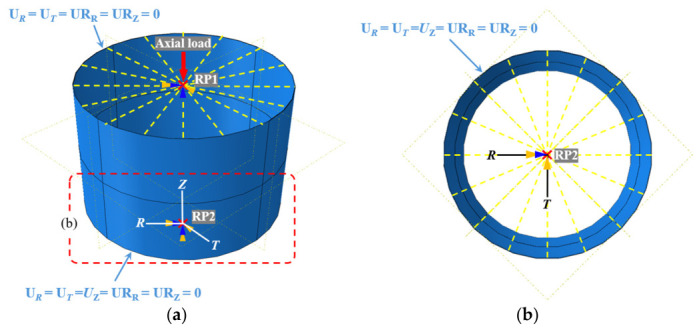
FEM solid model for the Eigen Value buckling analysis of bimodular thin cylindrical shell under axial compression. (**a**) Front of view and (**b**) bottom of view.

Based on the parameters in Table 1 and Table 2, we can conduct numerical simulations under five different modulus ratio combinations. It is worth noting that, in the numerical simulation of the Eigen Value buckling problem of bimodular axially compressed thin cylindrical shells, to eliminate the influence of local load effects, the common practice is to create a coupling constraint in the Interaction module, which constrains the displacement of the cylindrical shell edge to the reference point at the center of the circle, as shown in Figure 8. The yellow dashed line in Figure 8 represents the coupling constraint, which can synchronize the specified displacement of the shell edge to the reference points RP1 and RP2. Furthermore, to facilitate the constraint of displacement in the specified direction, we establish a local *R* −*T* − Z cylindrical coordinate system at the bottom of the shell, where *R*, *T*, and *Z* represent the radial, tangential, and axial directions of the shell, respectively. After setting the boundary conditions, a unit force with a magnitude of 1 is applied to the top of the shell, and the output Eigen Value is exactly the linear critical load of the shell.

**Figure 9:**
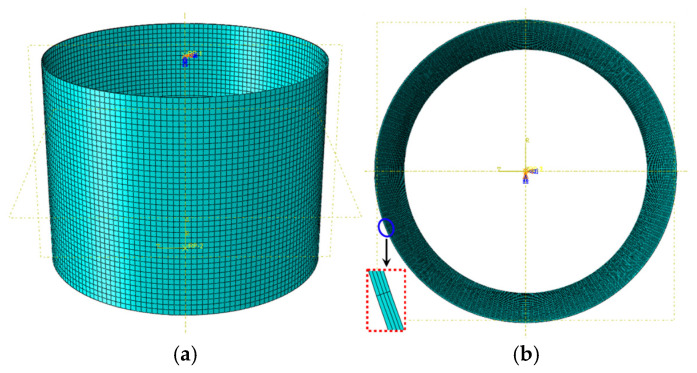
Schematic diagram of mesh division for the Eigen Value buckling analysis of the shell. (**a**) Front of view and (**b**) bottom of view.

For the meshing of this model, since the UMAT subroutine of the bimodular material used in this work is based on the Jaumann rate for stress update, the element type adopted for the Eigen Value buckling analysis of the shell is C3D20R. With regard to the selected element type, there is no need for overly dense mesh discretization, and four element layers laid out through the wall thickness are capable of delivering acceptable computational accuracy. When direct meshing is implemented on the solid cylindrical shell model in the simulation, simply boosting the absolute mesh density fails to effectively improve the computational precision as expected. This can be attributed to the fact that a monotonous growth in element quantity not only increases the computational burden and extends the solving duration, but also triggers element distortion on the curved geometry of the cylindrical shell. To address this issue, the solid cylindrical shell model is processed via datum cutting in this work, which effectively guarantees the shape regularity of elements during meshing and yields sound computational results, as depicted in Figure 9. Moreover, the geometric parameters of the shell model employed herein are consistent with those in Reference [30], which has elaborated on the meshing principles for axially compressed bimodular thin cylindrical shells in detail. Accordingly, the specific arrangement of mesh sizing is not further described in the current manuscript.

Subsequently, based on the above numerical model, we compare the linear critical loads obtained from the simplified mechanical model of this work, the pure axial compression solution proposed by Jones, and the finite element solution, and present the corresponding errors between these solutions. According to the values of geometric parameters in Table 2, the calculation results of these linear critical loads are presented in four subsections in ascending order of the radius-to-thickness ratio of the shells, and the displacement contours calculated by ABAQUS and their corresponding Eigen Values are provided in the corresponding subsections.

### 5.3. Example of R/t = 100

#### 5.3.1. For *L* = 200 mm

**Figure 10:**
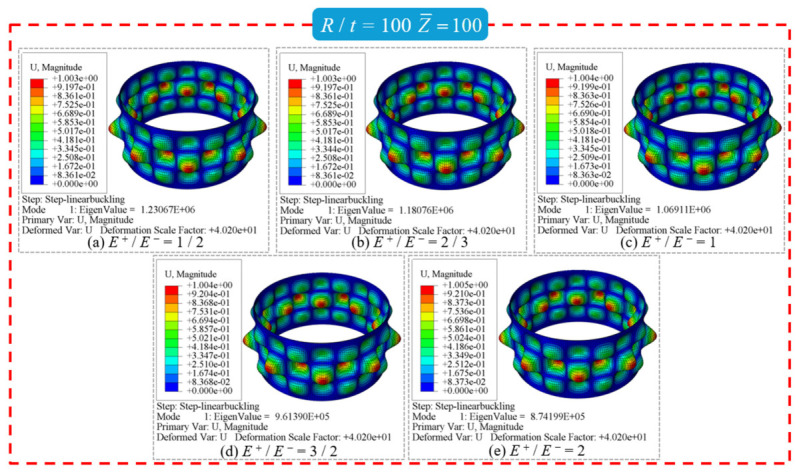
Displacement contours and corresponding Eigen Values under different *E*^+^/*E*^−^ ratios when $\bar{Z}$ = 100.

We first consider the linear critical load of the shell with a height of 200 mm and a *R*/*t* ratio of 100, where the geometric parameter $\bar{Z}$ of the shell is 100. With different combinations of *E*^+^/*E*^−^ ratio in Table 1, we can calculate a series of corresponding critical loads. The calculation results of each theoretical solution and numerical solution are listed in Table 3, and the error analysis between the theoretical solution of this study and other solutions is presented in Table 4. Figure 10 shows the displacement contours of the finite element results and their corresponding Eigen Values under different modulus ratios. It is worth noting that the Eigen Value calculated by the finite element method is actually the critical concentrated force *F^e^_cr_* applied at the center of the top of the shell. We need to convert the Eigen Values *F^e^_cr_* into *q^e^_cr_* through the relation *q^e^_cr_* = *F^e^_cr_*/(2*πRt*) before putting it into Table 3 for comparison. The same processing method is adopted for all subsequent *R*/*t* cases, so it will not be repeated hereinafter in the follow-up analysis.

#### 5.3.2. For *L* = 300 mm

**Figure 11:**
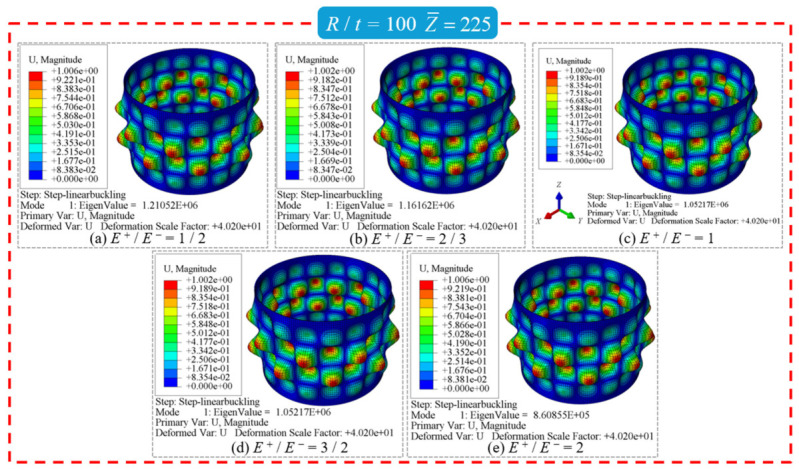
Displacement contours and corresponding Eigen Values under different *E*^+^/*E*^−^ ratios when $\bar{Z}$ = 225.

When the shell height is 300 mm and the *R*/*t* ratio remains 100, the geometric parameter $\bar{Z}$ of the shell is 225. Since the expressions of each theoretical solution do not contain the shell height *L*, the change of *L* will not affect the theoretical calculation results of the linear critical load of the shell. However, the finite element solution is affected by the change of shell height, so Table 5 only presents the finite element calculation results under different shell heights and their corresponding errors. Furthermore, Table 6 also presents the error between the theoretical solution of this work and the finite element results when *L* is changed to 300 mm. Meanwhile, Figure 11 also shows the displacement contours of the finite element results and their corresponding Eigen Values of the bimodular axially compressed thin cylindrical shells under this *R*/*t* ratio when *L*=300 mm.

### 5.4. Example of R/t = 125

#### 5.4.1. For *L* = 200 mm

**Figure 12:**
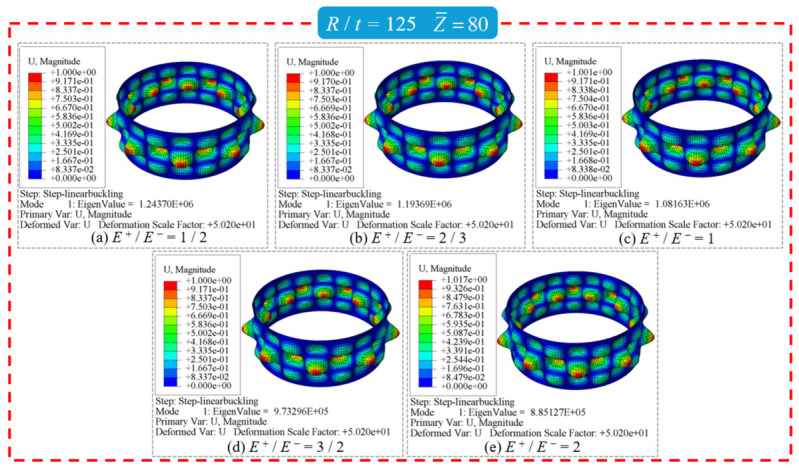
Displacement contours and corresponding Eigen Values under different *E*^+^/*E*^−^ ratios when $\bar{Z}$ = 80.

Consider the linear critical load of the shell with a height of 200 mm and a *R*/*t* ratio of 125, where the geometric parameter $\bar{Z}$ of the shell is 80. In this case, Table 7 presents a series of critical load values under different *E*^+^/*E*^−^ ratios, and Table 8 presents the error analysis between the theoretical solution of this study and other solutions. Figure 12 shows the displacement contours of the finite element results and their corresponding Eigen Values under different *E*^+^/*E*^−^ ratios.

#### 5.4.2. For *L* = 300 mm

**Figure 13:**
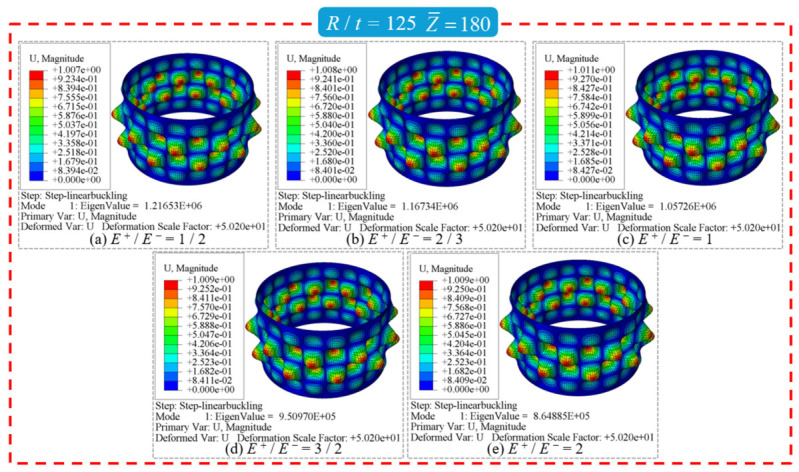
Displacement contours and corresponding Eigen Values under different *E*^+^/*E*^−^ ratios when $\bar{Z}$ = 180.

Consider the linear critical load of the shell with a height of 300 mm and a *R*/*t* ratio that remains 125, where the geometric parameter $\bar{Z}$ of the shell is 180. In this case, Table 9 presents the finite element solution under different shell heights and their corresponding errors, and Table 10 presents the error between the theoretical solution of this study and the finite element solution. Figure 13 shows the finite element displacement contours and their corresponding Eigen Values when *L* = 300 mm and *R*/*t* = 125.

### 5.5. Example of R/t = 200

#### 5.5.1. For *L* = 200 mm

**Figure 14:**
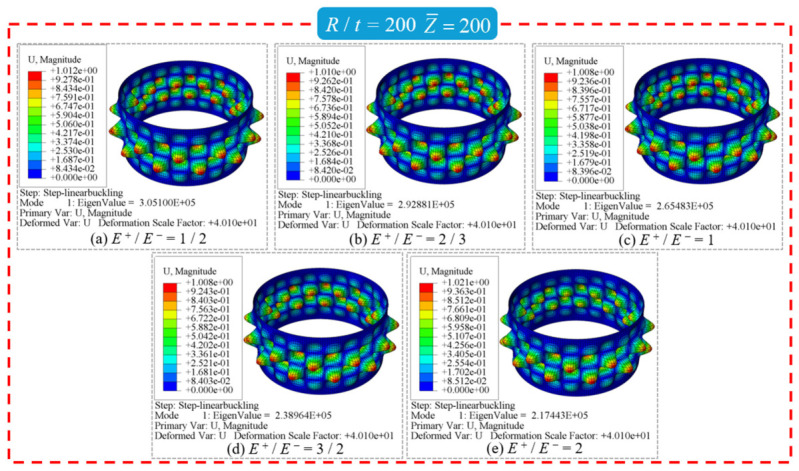
Displacement contours and corresponding Eigen Values under different *E*^+^/*E*^−^ ratios when $\bar{Z}$ = 200.

Consider the linear critical load of the shell with a height of 200 mm and a *R*/*t* ratio of 200, where the geometric parameter $\bar{Z}$ of the shell is 200. In this case, Table 11 presents a series of critical load values under different *E*^+^/*E*^−^ ratios, and Table 12 presents the error analysis between the theoretical solution of this study and other solutions. Figure 14 shows the displacement contours of the finite element results and their corresponding Eigen Values under different *E*^+^/*E*^−^ ratios.

#### 5.5.2. For *L* = 300 mm

**Figure 15:**
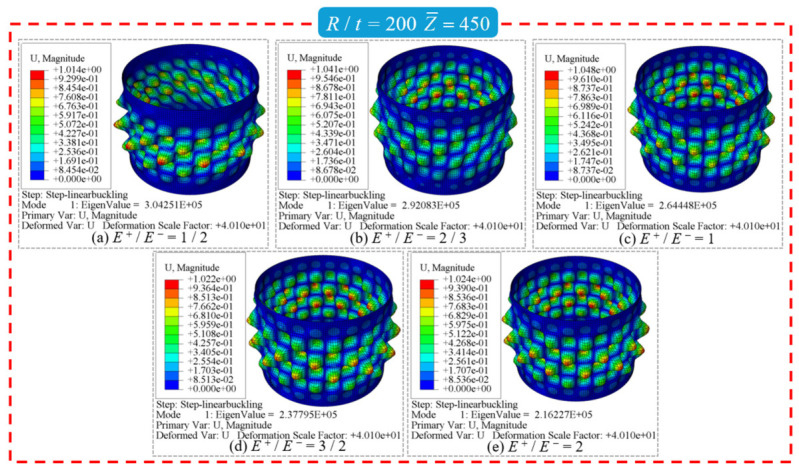
Displacement contours and corresponding Eigen Values under different *E*^+^/*E*^−^ ratios when $\bar{Z}$ = 450.

Consider the linear critical load of the shell with a height of 300 mm and a *R*/*t* ratio that remains 200, where the geometric parameter $\bar{Z}$ of the shell is 450. In this case, Table 13 presents the finite element solution under different shell heights and their corresponding errors, and Table 14 presents the error between the theoretical solution of this study and the finite element solution. Figure 15 shows the finite element displacement contours and their corresponding Eigen Values when *L* = 300 mm and *R*/*t* = 200.

### 5.6. Example of R/t = 250

#### 5.6.1. For *L* = 200 mm

**Figure 16:**
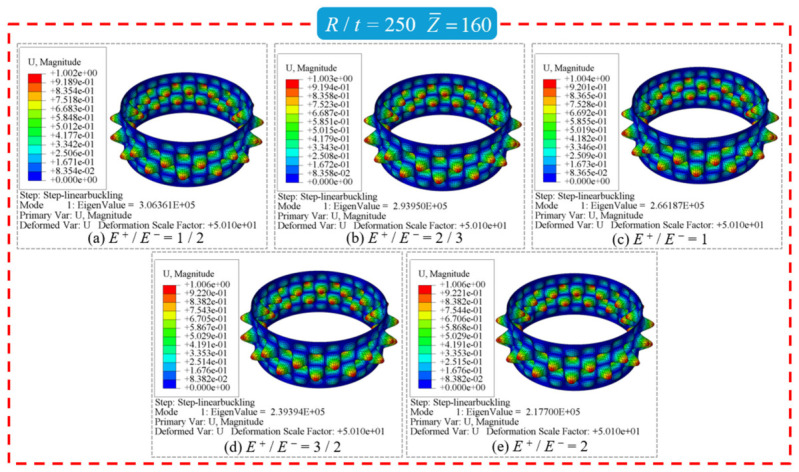
Displacement contours and corresponding Eigen Values under different *E*^+^/*E*^−^ ratios when $\bar{Z}$ = 160.

Consider the linear critical load of the shell with a height of 200 mm and a *R*/*t* ratio of 250, where the geometric parameter $\bar{Z}$ of the shell is 160. In this case, Table 15 presents a series of critical load values under different *E*^+^/*E*^−^ ratios, and Table 16 presents the error analysis between the theoretical solution of this study and other solutions. Figure 16 shows the displacement contours of the finite element results and their corresponding Eigen Values under different *E*^+^/*E*^−^ ratios.

#### 5.6.2. For *L* = 300 mm

**Figure 17:**
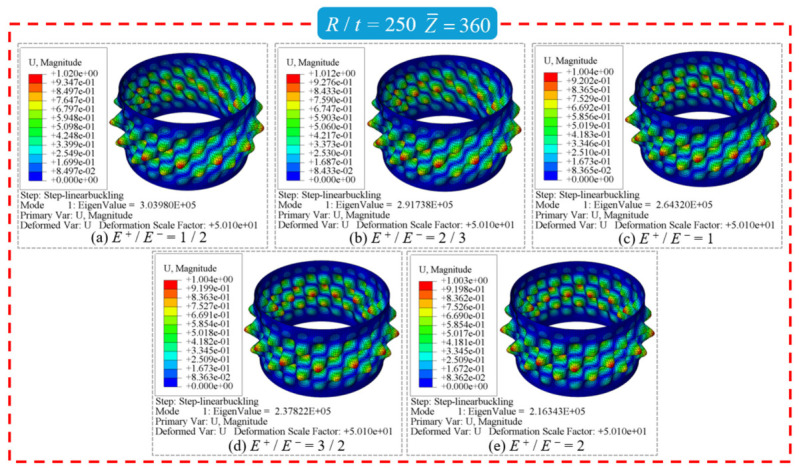
Displacement contours and corresponding Eigen Values under different *E*^+^/*E*^−^ ratios when $\bar{Z}$ = 360.

Consider the linear critical load of the shell with a height of 300 mm and a *R*/*t* ratio that remains 250, where the geometric parameter $\bar{Z}$ of the shell is 360. In this case, Table 17 presents the finite element solution under different shell heights and their corresponding errors, and Table 18 presents the error between the theoretical solution of this study and the finite element solution. Figure 17 shows the finite element displacement contours and their corresponding Eigen Values when *L* = 300 mm and *R*/*t* = 250.

In summary, it can be easily observed from all the above figures and tables that although there are differences between the numerical simulations and the theoretical solutions, these differences are within the acceptable range. The validity of the theoretical solution of this work and the reasons for the above differences will be analyzed in the next section.

## 6. Results and Discussion

In Section 5, based on the material parameters in Table 1 and the geometric parameters in Table 2, we obtained the numerical calculation results of the small-deflection theoretical solution and the FEM solution when *R*/*t* equals 100, 125, 200, and 250, respectively. In this section, taking these calculation results as the reference, we first verify the validity of the theoretical solution of the linear critical load in this study, and then discuss the influence of the material’s bimodular effect on the linear critical load of the shell.

### 6.1. Validation of the Theoretical Solution

By observing the data in Table 3, Table 4, Table 5, Table 6, Table 7, Table 8, Table 9, Table 10, Table 11, Table 12, Table 13, Table 14, Table 15, Table 16, Table 17 and Table 18 and Figure 10, Figure 11, Figure 12, Figure 13, Figure 14, Figure 15, Figure 16 and Figure 17, it is not difficult to find the following phenomena:

(i) Compared with the linear critical load theoretical solution proposed by Jones, the theoretical solution in our study is closer to the FEM solution than Jones solution when the modulus ratio satisfies *E*^+^/*E*^−^ > 1; however, when the modulus ratio satisfies *E*^+^/*E*^−^ < 1, Jones theoretical solution is closer to the FEM solution, and the gap between it and the FEM solution is smaller. In particular, when *E*^+^/*E*^−^ = 1, the calculation results of Jones theoretical solution and the theoretical solution of this study are always consistent.

(ii) For the FEM solution, under the condition of the same *R*/*t* ratio of the shell, the critical load when the shell height *L* = 300 mm is generally reduced compared with that when *L* = 200 mm. Moreover, the smaller the *R*/*t* ratio of the shell, the more obvious the overall reduction in the critical load is. That is, under the same *R*/*t* ratio, the Eigen Value obtained from the finite element model of the shell with a height of *L*=300 mm is closer to the theoretical solution of this study than that with a height of *L* = 200 mm. Furthermore, the smaller the geometric parameter $\bar{Z}$ of the shell is, the more obvious the reduction in the Eigen Value caused by the increase in height is.

Through the above phenomena, we can make the following discussion on the validity of the theoretical solution of this work

From phenomenon (i), it can be seen that although Jones solution is closer to the FEM solution than the theoretical solution of this study when the modulus ratio satisfies *E*^+^/*E*^−^ < 1, for the case of *E*^+^/*E*^−^ > 1, the maximum difference between Jones theoretical solution and the FEM solution can reach about 8% to 10%. However, the maximum difference between the theoretical solution in our work and the FEM solution is only about 6%, and the error values are basically symmetrically distributed with respect to the case of *E*^+^/*E*^−^ = 1. That is, the theoretical solution of this work is closer to the FEM solution on the whole. Therefore, from this aspect, it can be illustrated that the assumption proposed in our study that the circumferential membrane stress of the shell is always compressive before and after buckling is reasonable, and the calculation formula of the linear critical load of the shell, i.e., Equation (42), is also valid. Furthermore, when *E*^+^/*E*^−^ = 1, Jones theoretical solution is always consistent with the theoretical solution of our work. This phenomenon illustrates that when the bimodular effect of the material is not considered, the two solutions can both reduced to the same expression. If we substitute the reduced conditions *E*^+^ = *E*^−^ = *E* and *μ*^+^ = *μ*^−^ = *μ* into Equations (18), (42), and (43), the unified reduced solution can be obtained as follows

(49) $${\bar{q}}_{cr}=\frac{E}{\sqrt{3(1-\mu^{2})}}\frac{t}{R},$$

which is exactly the well-known classical calculation formula of the linear critical load for axially compressed thin cylindrical shells. The inversion property of above solutions also indirectly proves the validity of the theoretical solution of this study.

Furthermore, from phenomenon (ii), we can also know that when the value of the geometric parameter $\bar{Z}$ of the shell is small, appropriately increasing the height of the shell can make the FEM solution closer to the theoretical solution of our study. For example, for the case of *R*/*t* = 125, the maximum error between the FEM solution and the theoretical solution of this work when the shell height *L* = 200 mm is 6.262%, but when the shell height is increased to *L* = 300 mm, the maximum error is reduced to 4.068%. That is, the calculation results of the theoretical solution of our study are actually more suitable for the prediction of the linear critical load of relatively slender shells. This view can also be obtained by observing all the numerical examples of shells with larger $\bar{Z}$ values in Section 5. It can be seen from Table 13 that when $\bar{Z}$ = 450 (the maximum in the above examples), the influence of the increase in shell height on the Eigen Value is within 0.6%. This phenomenon is consistent with the view in the classical shell stability theory that Equation (49) is only applicable to moderately long shells. Therefore, this view in the classical shell stability theory is also applicable to the calculation formula of the linear critical load of the bimodular axially compressed thin cylindrical shells.

In summary, the above analysis by comparing Jones theoretical solution, the theoretical solution of this work, and the FEM solution, illustrates the rationality of the simplified mechanical model in our study and the validity of the small-deflection theoretical solution of this work. Moreover, the above analysis results show that, just like the classical shell stability theory, for the bimodular axially compressed thin cylindrical shells with moderate length, the calculation formula of the linear critical load proposed in our study is sufficiently accurate. However, for shells that are too slender or too shorter and squatter, the theoretical solution in our study may produce large errors compared with the FEM solution. Meanwhile, it should be noted that Jones theoretical research is strictly based on Ambartsumyan’s original constitutive model of bimodular materials based on the sign criterion of principal stress, while the theoretical solution in our study is based on the tensile-compressive subarea simplified mechanical model shown in Figure 5. This inconsistency of the constitutive model will also lead to large differences between the two theoretical solutions. Nevertheless, from the results of the analysis of the above numerical examples, compared with the theoretical solution from Jones, the theoretical solution of our study is obviously closer to the ABAQUS simulation results on the whole.

Notably, the simplified mechanics model in this paper is established based on the small-deflection linear buckling assumption for perfect shells, and therefore cannot cover nonlinear buckling scenarios with initial geometric imperfections. The actual buckling of imperfect shells presents as limit point buckling, with its load-bearing capacity significantly lower than the critical load of ideal linear buckling. Hence, the analytical results of this work can only serve as the theoretical upper bound of the ideal load-bearing capacity, leading to conservative predictions. Accordingly, the findings of this study are only applicable to the preliminary design and conservative load capacity estimation of medium-length perfect bimodular thin cylindrical shells, or can be incorporated as an initial imperfection baseline into the post-buckling analysis of bimodular axially compressed thin cylindrical shells. For practical engineering components with imperfections, the results shall be modified in combination with imperfection sensitivity analysis and nonlinear post-buckling investigations.

### 6.2. Influence of the Bimodular Effect on the Buckling Behavior

**Figure 18:**
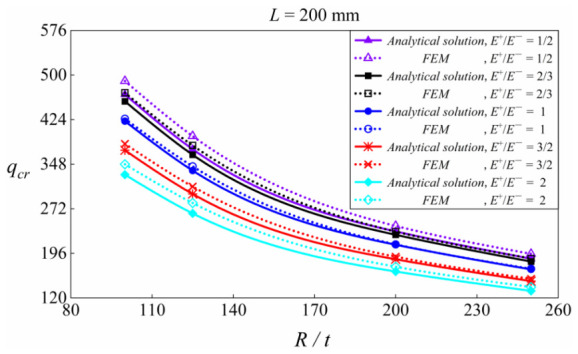
Influence of bimodular effect on the present analytical solution of linear critical load and the corresponding FEM solution.

**Figure 19:**
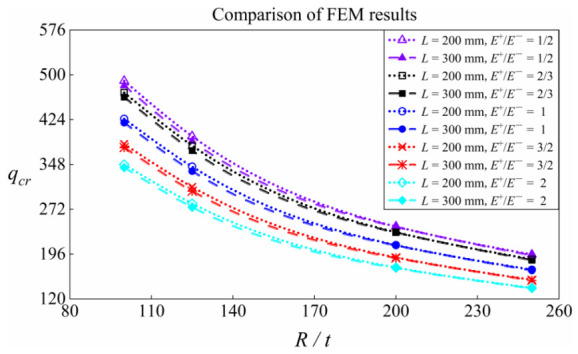
Influence of bimodular effect on the FEM simulation results for the shell with different heights.

From the data in Table 3, Table 4, Table 5, Table 6, Table 7, Table 8, Table 9, Table 10, Table 11, Table 12, Table 13, Table 14, Table 15, Table 16, Table 17 and Table 18 as well as Figure 10, Figure 11, Figure 12, Figure 13, Figure 14, Figure 15, Figure 16 and Figure 17, we can further analyze how the introduction of bimodular effect influences the linear critical load and buckling mode of shells with different geometrical dimensions. To more intuitively illustrate the variation trend of the shell’s linear critical load with the *R*/*t* ratio, we plotted the data from Table 3, Table 4, Table 5, Table 6, Table 7, Table 8, Table 9, Table 10, Table 11, Table 12, Table 13, Table 14, Table 15, Table 16, Table 17 and Table 18 in Figure 18 and Figure 19. Through Figure 18 and Figure 19 and the ABAQUS displacement contours presented in Section 5, it is not difficult to draw the following conclusions:

(i) Figure 18 uses a shell height *L* = 200 mm as an example, presenting the variation trend of the FEM solution and the present analytical solution with the *R*/*t* ratio under different *E*^+^/*E*^−^ ratios. It can be seen that for both the FEM solution and the present theoretical solution, when the modulus ratio *E*^+^/*E*^−^ > 1, the critical load of the shell is more sensitive to the bimodular effect of materials; when *E*^+^/*E*^−^ < 1, the influence of the bimodular effect of materials on the shell’s critical load is relatively small.

(ii) Figure 19 presents the variation trend of the shell’s linear critical load obtained from FEM with the *R*/*t* ratio under different shell heights and modulus ratios. It can be learned from this figure that only when the *R*/*t* ratio of the shell *R*/*t* ≤ 180 does the difference in linear critical load between shells of different heights caused by the bimodular effect of materials gradually become noticeable. In other words, when *R/t* ≥ 180 and the variation range of the shell height is small, the influence of height variation on the shell’s linear critical load can be neglected.

(iii) From the series of Eigen Value buckling displacement contours presented in Section 5, it can be seen that for a fixed combination of *R*/*t* ratio and shell height, the variation of the *E*^+^/*E*^−^ will not change the axial and circumferential buckling half-wave numbers *m* and *n* of the shell during linear buckling. Furthermore, from Figure 18 and Figure 19, it can be further learned that when the shell height *L* = 200 mm, regardless of the *R*/*t* ratio, the maximum transverse bending deflection of the shell during buckling reaches its peak when *E*^+^/*E*^−^ = 2, and reaches its minimum when *E*^+^/*E*^−^ = 1/2; whereas when the shell height *L* = 300 mm, the situation is slightly different, and the peak of the shell’s transverse bending deflection does not necessarily occur when the *E*^+^/*E*^−^ ratio reaches its extreme value.

In summary, from conclusion (i), it can be seen that for both the FEM solution and the analytical solution, the weakening effect of the bimodular effect on the shell’s linear critical load is greater than its corresponding enhancing effect. Specifically, this phenomenon becomes more pronounced when the *R*/*t* ratio of the shell is small. Meanwhile, from conclusion (ii), when considering the influence of shell height on the linear critical load via the FEM solution, for shells with a large *R*/*t* ratio (*R/t* ≥ 180), the influence caused by the variation of shell height can be neglected to a certain extent. This holds true for both the classical small-deflection solution and the small-deflection solution considering the bimodular effect of materials. Finally, from conclusion (iii) and Figure 10, Figure 11, Figure 12, Figure 13, Figure 14, Figure 15, Figure 16 and Figure 17, we can clearly observe that although the bimodular effect of materials cannot change the axial and circumferential half-wave numbers *m* and *n* of the shell during Eigen Value buckling, it can change the amplitude of the transverse bending deflection during buckling. This indicates that the bimodular effect of material can alter the shell’s lateral bending stiffness, and thus influence the shell’s linear critical load. Meanwhile, in view of the fundamental principles of linear buckling, the buckling mode morphology of thin cylindrical shells—as reflected by the distribution characteristics of axial and circumferential half-wave numbers—is governed by the shell’s geometric parameters and boundary conditions, and corresponds to the deformation pattern that minimizes the total potential energy of the system at the onset of instability. The bimodular effect uniformly modifies the membrane stiffness and bending stiffness of the shell without introducing spatial inhomogeneity in stiffness. As a result, it does not alter the energy-optimal buckling waveform, but only adjusts the critical load and the amplitude of transverse deflection through the variation of the overall structural stiffness. Furthermore, the maximum transverse bending deflection of the shell depends not only on the bimodular effect of materials, but also on the shell’s height.

## 7. Conclusions

In this study, we theoretically analyzed the linear buckling problem of bimodular thin cylindrical shells under uniform axial compression load, and obtained the corresponding theoretical solution for the critical load. The numerical simulation based on ABAQUS has also verified the validity of the present simplified mechanical model and the theoretical solution. We have discussed in detail the influence of various important parameters on the shell’s linear critical load; these parameters include the *E*^+^/*E*^−^ ratio from the material aspect, as well as the *R*/*t* ratio, the shell height *L*, and the shell’s $\bar{Z}$ parameter from the geometrical aspect. The following conclusions can be drawn.

1. Incorporating the bimodular behavior of a material substantially alters the linear critical load of thin cylindrical shells under axial compression. Essentially, the bimodular effect governs the linear buckling response by modifying the lateral bending stiffness of the shell. Hence, a suitable distribution of tensile and compressive moduli can effectively improve the load-carrying capacity of axially compressed thin cylindrical shells.
2. Since the expression of the linear critical load derived from the present simplified mechanical model does not contain the shell height *L*, the present theoretical solution, is only applicable to the critical load prediction of moderately long shells. Only when the *R*/*t* ratio of the shell (*R*/*t* ≥ 180) can the influence of shell height variation be neglected to a certain extent.
3. The *R*/*t* ratio of the shell exerts a significant influence on the linear buckling behavior of bimodular axially compressed thin cylindrical shells. The smaller the *R*/*t* ratio of the shell, the more sensitive its linear critical load is to the bimodular effect. Therefore, it is essential to account for the bimodular effect of materials during the design of shells with a small *R*/*t* ratio.
4. For the linear buckling behavior of axially compressed thin cylindrical shells, the introduction of the bimodular effect of materials will not change the buckling mode of the shell, but will influence its maximum transverse bending deflection. Furthermore, under different *E*^+^/*E*^−^ ratios, the maximum transverse bending deflection of the shell depends on both its *R*/*t* ratio and the shell height *L*.

In summary, the results obtained in this study are generally consistent with the core mechanical assumptions underlying the proposed analytical model. Both the continuously compressive state of the circumferential membrane stress and the invariance of the buckling mode with changes in modulus ratio are supported by evidence from numerical post-processing. The maximum discrepancy between the theoretically predicted critical loads and the finite element results is within 6%, which arises from the assumption of idealized decoupling between membrane stress and bending stress. In addition, the parametric variation trends of buckling performance also conform to the expected physical principles.

From a practical engineering standpoint, the solution in present study is suitable for preliminary bearing capacity estimation of medium-length thin shells with minor initial imperfections; it requires targeted correction when applied to extremely long shells prone to Eulerian global buckling or short shells dominated by boundary constraint effects. Given that initial geometric imperfections will degrade the actual buckling capacity of shell structures, the ideal solution derived in this work serves as the theoretical upper bound of bearing capacity, and the imperfection sensitivity of bimodular shells remains to be further quantified. For subsequent experimental validation, it is recommended to select typical bimodular cast alloys such as ZA27 as test materials, calibrate tensile and compressive elastic moduli with test error controlled within 2% via standard uniaxial tests, and carry out axial compression buckling tests on shell specimens with pre-defined imperfection amplitudes to systematically verify the model performance. For instance, standard uniaxial tensile specimens and compressive specimens can be fabricated from the same batch of bimodular material, conforming to the geometric specifications of ASTM E8/E8M [63] and ASTM E9 standards [64], respectively. Quasi-static monotonic loading tests will be performed to acquire complete stress–strain curves. Through linear regression of the linear elastic portion, the tensile elastic modulus *E*^+^, the compressive elastic modulus *E*^−^, and the corresponding Poisson’s ratios *μ^+^* and *μ^−^* can be determined. On this basis, axial compression buckling tests on full-scale cylindrical shell specimens can be conducted to systematically validate the accuracy and applicability of the analytical solution proposed in this work. Finally, we would also like to point out that the solution of many important problems needs to be based on the linear small-deflection solution. For example, in the existing postbuckling analysis of thin cylindrical shells under axial compression, the linear buckling mode of the shell is usually introduced as an imperfection. Therefore, based on the research of this paper, the postbuckling behavior analyze of bimodular axially compressed thin cylindrical shells with imperfection can be taken as a possible future research direction.

## Figures and Tables

**Table 1:** Tensile-compressive elastic moduli and Poisson’s ratio under different *E*^+^/*E*^−^ combinations [30].

*E*^+^/*E*^−^	*E*^+^ (GPa)	*E*^−^ (GPa)	*μ* ^+^	*μ* ^−^
1/2	45.93	91.87	0.22	0.44
2/3	55.12	82.68	0.264	0.396
1	68.9	68.9	0.33	0.33
3/2	82.68	55.12	0.396	0.264
2	91.87	45.93	0.44	0.22

**Table 2:** Value combinations of each geometric parameter of the shells.

*L* (mm)	*R* (mm)	*t* (mm)	*L/R*	*R/t*	$\bar{Z}$
200	200	1	1	200	200
		2		100	100
	250	1	0.8	250	160
		2		125	80
300	200	1	1.5	200	450
		2		100	225
	250	1	1.2	250	360
		2		125	180

**Table 3:** Calculation results of each theoretical solution and finite element solution under different *E*^+^/*E*^−^ ratios when $\bar{Z}$ = 100.

*E*^+^/*E*^−^	*L* (mm)	Critical Load *q_cr_* (MPa)		
		Analytical Solution (Jones [62])	Analytical Solution (This Study)	FEM Solution
1/2	200	447.805	466.872	489.668
2/3		443.799	455.131	469.809
1		421.401	421.401	425.385
3/2		380.812	371.613	382.525
2		344.013	330.128	347.833

**Table 4:** Error analysis between the present theoretical solution and other solutions when $\bar{Z}$ = 100.

*E*^+^/*E*^−^	*L* (mm)	Error (%)	
		Comparison with Jones Solution	Comparison with FEM Solution
1/2	200	4.258	4.655
2/3		2.553	3.124
1		0.000	0.937
3/2		2.416	2.853
2		4.036	5.090

**Table 5:** Comparison of the FEM solutions for shells with different heights when *R*/*t* = 100.

*E*^+^/*E*^−^	Critical Load *q_cr_* (MPa)		Error (%)
	*L* = 300 mm	*L* = 200 mm	
1/2	481.651	489.668	1.664
2/3	462.194	469.809	1.648
1	418.645	425.385	1.610
3/2	376.591	382.525	1.576
2	342.523	347.833	1.550

**Table 6:** Error analysis between the present solution and FEM solution for different *E*^+^/*E*^−^ ratios when $\bar{Z}$ = 225.

*E*^+^/*E*^−^	Critical Load *q_cr_* (MPa)		Error (%)
	Analytical Solution (This Study)	FEM Solution	
1/2	466.872	481.651	3.068
2/3	455.131	462.194	1.528
1	421.401	418.645	0.658
3/2	371.613	376.591	1.322
2	330.128	342.523	3.619

**Table 7:** Calculation results of each theoretical solution and finite element solution under different *E*^+^/*E*^−^ ratios when $\bar{Z}$ = 80.

*E*^+^/*E*^−^	*L* (mm)	Critical Load *q_cr_* (MPa)		
		Analytical Solution (Jones [62])	Analytical Solution (This Study)	FEM Solution
1/2	200	358.244	373.498	395.882
2/3		355.040	364.105	379.963
1		337.121	337.121	344.294
3/2		304.650	297.290	309.810
2		275.211	264.103	281.745

**Table 8:** Error analysis between the present theoretical solution and other solutions when $\bar{Z}$ = 80.

*E*^+^/*E*^−^	*L* (mm)	Error (%)	
		Comparison with Jones Solution	Comparison with FEM Solution
1/2	200	4.258	5.654
2/3		2.553	4.174
1		0.000	2.083
3/2		2.416	4.041
2		4.036	6.262

**Table 9:** Comparison of the FEM solutions for shells with different heights when *R*/*t* = 125.

*E*^+^/*E*^−^	Critical Load *q_cr_* (MPa)		Error (%)
	*L* = 300 mm	*L* = 200 mm	
1/2	387.234	395.882	2.233
2/3	371.576	379.963	2.257
1	336.536	344.294	2.305
3/2	302.703	309.810	2.348
2	275.301	281.745	2.341

**Table 10:** Error analysis between the present solution and FEM solution for different *E*^+^/*E*^−^ ratios when $\bar{Z}$ = 180.

*E*^+^/*E*^−^	Critical Load *q_cr_* (MPa)		Error (%)
	Analytical Solution (This Study)	FEM Solution	
1/2	373.498	387.234	3.547
2/3	364.105	371.576	2.011
1	337.121	336.536	0.174
3/2	297.290	302.703	1.788
2	264.103	275.301	4.068

**Table 11:** Calculation results of each theoretical solution and finite element solution under different *E*^+^/*E*^−^ ratios when $\bar{Z}$ = 200.

*E*^+^/*E*^−^	*L* (mm)	Critical Load *q_cr_* (MPa)		
		Analytical Solution (Jones [62])	Analytical Solution (This Study)	FEM Solution
1/2	200	223.903	233.436	242.791
2/3		221.900	227.565	233.067
1		210.700	210.700	211.265
3/2		190.406	185.806	190.162
2		172.007	165.064	173.036

**Table 12:** Error analysis between the present theoretical solution and other solutions when $\bar{Z}$ = 200.

*E*^+^/*E*^−^	*L* (mm)	Error (%)	
		Comparison with Jones Solution	Comparison with FEM Solution
1/2	200	4.258	3.853
2/3		2.553	2.361
1		0.000	0.267
3/2		2.416	2.291
2		4.036	4.607

**Table 13:** Comparison of the FEM solutions for shells with different heights when *R*/*t* = 200.

*E*^+^/*E*^−^	Critical Load *q_cr_* (MPa)		Error (%)
	*L* = 300 mm	*L* = 200 mm	
1/2	242.115	242.791	0.279
2/3	232.432	233.067	0.273
1	210.441	211.265	0.392
3/2	189.231	190.162	0.492
2	172.068	173.036	0.563

**Table 14:** Error analysis between the present solution and FEM solution for different *E*^+^/*E*^−^ ratios when $\bar{Z}$ = 450.

*E*^+^/*E*^−^	Critical Load *q_cr_* (MPa)		Error (%)
	Analytical Solution (This Study)	FEM Solution	
1/2	233.436	242.115	3.585
2/3	227.565	232.432	2.094
1	210.700	210.441	0.123
3/2	185.806	189.231	1.810
2	165.064	172.068	4.070

**Table 15:** Calculation results of each theoretical solution and finite element solution under different *E*^+^/*E*^−^ ratios when $\bar{Z}$ = 160.

*E*^+^/*E*^−^	*L* (mm)	Critical Load *q_cr_* (MPa)		
		Analytical Solution (Jones [62])	Analytical Solution (This Study)	FEM Solution
1/2	200	179.122	186.749	195.035
2/3		177.520	182.052	187.134
1		168.560	168.560	169.460
3/2		152.325	148.645	152.427
2		137.605	132.051	138.592

**Table 16:** Error analysis between the present theoretical solution and other solutions when $\bar{Z}$ = 160.

*E*^+^/*E*^−^	*L* (mm)	Error (%)	
		Comparison with Jones Solution	Comparison with FEM Solution
1/2	200	4.258	4.248
2/3		0.000	0.531
1		2.416	2.481
3/2		4.036	4.720
2		0.000	0.531

**Table 17:** Comparison of the FEM solutions for shells with different heights when *R*/*t* = 250.

*E*^+^/*E*^−^	Critical Load *q_cr_* (MPa)		Error (%)
	*L* = 300 mm	*L* = 200 mm	
1/2	242.115	242.791	0.279
2/3	232.432	233.067	0.273
1	210.441	211.265	0.392
3/2	189.231	190.162	0.492
2	172.068	173.036	0.563

**Table 18:** Error analysis between the present solution and FEM solution for different *E*^+^/*E*^−^ ratios when $\bar{Z}$ = 360.

*E*^+^/*E*^−^	Critical Load *q_cr_* (MPa)		Error (%)
	Analytical Solution (This Study)	FEM Solution	
1/2	233.436	242.115	3.585
2/3	227.565	232.432	2.094
1	210.700	210.441	0.123
3/2	185.806	189.231	1.810
2	165.064	172.068	4.070

## Data Availability

The original contributions presented in this study are included in the article. Further inquiries can be directed to the corresponding author.
